# Antidepressant outcomes of high-frequency repetitive transcranial magnetic stimulation (rTMS) with F8-coil and deep transcranial magnetic stimulation (DTMS) with H1-coil in major depression: a systematic review and meta-analysis

**DOI:** 10.1186/s12888-019-2106-7

**Published:** 2019-05-07

**Authors:** Helena M. Gellersen, Karina Karolina Kedzior

**Affiliations:** 1 Department of Psychology, University of Cambridge, Cambridge, UK; 2 Faculty of Social Sciences, City University of Applied Sciences, Bremen, Germany

**Keywords:** Repetitive transcranial magnetic stimulation (rTMS), Figure-of-eight coil (F8-coil), Deep transcranial magnetic stimulation (DTMS), H1-coil, Major depressive disorder (MDD), Meta-analysis

## Abstract

**Background:**

The current study aims to systematically assess and compare the antidepressant outcomes of repetitive transcranial magnetic stimulation (rTMS) with the figure-of-eight (F8)-coil and deep transcranial magnetic stimulation (DTMS) with the H1-coil in studies matched on stimulation frequency in unipolar major depressive disorder (MDD).

**Methods:**

Electronic search of Medline and PsycInfo identified 19 studies with stimulation frequency of 18–20 Hz using F8-coil (*k* = 8 randomised sham-controlled trials, RCTs, *k* = 3 open-label; *n* = 168 patients) or H1-coil (*k* = 1 RCT, *k* = 7 open-label; *n* = 200). Depression severity (the primary outcome) and response/remission rates (the secondary outcomes) were assessed at session 10.

**Results:**

Effects pooled with random-effects meta-analysis showed a large reduction in depression severity, 29% response, and 15% remission rates after 10 sessions of active stimulation with either coil relative to baseline. Reduction in depression severity was greater in studies with younger patients using either coil. The comparison between coils showed a larger reduction in depression severity in H1-coil vs. F8-coil studies (independent of the study design or the concurrent pharmacotherapy) and a trend towards higher remission rates in F8-coil vs. H1-coils studies. These effects are based on a low volume of studies, are not controlled for placebo, and may not be clinically-relevant. The stimulation protocols differed systematically because stimulation was more focal but less intense (80–110% of the resting motor threshold, MT) in the F8-coil studies and less focal but more intense (120% MT) in the H1-coil studies. Two seizures occurred in the H1-coil studies relative to none in the F8-coil studies.

**Conclusion:**

When matched on frequency, the higher-intensity and less focal stimulation with the H1-coil reduces depression more than the lower-intensity and more focal stimulation with the F8-coil. Head-to-head trials should compare the antidepressant outcomes of F8-coil and H1-coil to identify the most optimal stimulation protocols for acute and longer-lasting efficacy.

**Electronic supplementary material:**

The online version of this article (10.1186/s12888-019-2106-7) contains supplementary material, which is available to authorized users.

## Background

Non-invasive brain stimulation methods are established as viable treatment options for major depressive disorder (MDD) resistant to pharmacotherapy. One of such most thoroughly investigated methods is the high-frequency repetitive transcranial magnetic stimulation (rTMS) of the left dorsolateral prefrontal cortex (DLPFC) commonly applied with a figure-of-eight (F8) coil. This treatment aims to ameliorate the hypoactivity of the DLPFC characteristic of MDD and has acute moderate to large antidepressant effects as well as acceptable tolerability [1–4]. Since 2008 rTMS has been approved for treatment-resistant unipolar MDD by the U.S. Food and Drug Administration (FDA).

One alternative to rTMS with the F8-coil is the deep transcranial magnetic stimulation (DTMS) with the Hesed-coil (H-coil). The H-coil was designed to induce the electrical fields at different locations around the surface of the head that have a common direction and presumably summate in the deep neural areas [5]. While there are several types of H-coils, the H1-coil is comparable to the F8-coil because it stimulates mostly the left DLPFC [6]. However, relative to the focal stimulation achieved with the F8-coil, the H1-coil is less focal [6]. A consistent reduction of symptoms in some substance use disorders indirectly suggests that the H-coils may target deeper reward pathways [7] although the issue of depth is not resolved yet. The literature so far suggests that the H1-coil produces consistent short-term antidepressant effects in MDD [8, 9] and is FDA-approved for treatment-resistant unipolar MDD since 2013.

Although rTMS with F8-coil and DTMS with H1-coil are promising treatments for unipolar MDD, the head-to-head comparisons in efficacy of both methods are lacking so far. A recent network meta-analysis of currently available non-invasive brain stimulation methods concluded that the high-frequency rTMS with F8-coil over the left DLPFC is among the most efficacious techniques, whereas DTMS with H1-coil is not more effective than sham [10]. However, since the analysis focused on the double-blind, randomised-controlled trials (RCT) with inactive sham groups, it compared only one available RCT using DTMS with H1-coil [9] relative to over 50 RCTs using rTMS with F8-coil and a plethora of different stimulation paradigms [10]. Consequently, a comparison in efficacy of both coils might have been affected by the imbalance in the volume of available evidence. Furthermore, there are differences in the FDA-approved protocols for the clinical application of the two devices. Inspection of the stimulation parameters in 54 RCTs with MDD patients [3] revealed that rTMS with F8-coil was most often applied with the intensity of 80–120% of the resting motor threshold (MT) and either 10 Hz for at least 10 daily sessions or 20 Hz in 10 sessions. In contrast, DTMS with H1-coil was always applied with the intensity of 120% MT and 18–20 Hz over 20 daily sessions in 10, mostly open-label, studies with MDD patients [8]. These differences in the stimulation protocols prevent any direct comparisons of the FDA-approved clinical paradigms of both coils in the treatment of MDD.

Similar to the network meta-analysis [10] the aim of the current study is to systematically assess and compare the antidepressant outcomes of rTMS with F8-coil and DTMS with H1-coil in MDD. However, since only one RCT was conducted with the DTMS method in MDD to date [9], we focus on the following parameters rather than study designs:

1. the same high-frequency of stimulation (18–20 Hz) because such frequencies were used in all DTMS studies to date,
2. the same antidepressant outcomes primarily focusing on the continuous measures that may be more appropriate to account for individual variability than the artificial classification into groups based on dichotomous measures typically used to quantify the clinical efficacy [3, 11],
3. the same time of outcome assessment (after 10 daily sessions relative to baseline) because rTMS with the frequency of 20 Hz was applied for 10 days only in most studies to date,
4. the same outcome assessment scale (Hamilton Depression Rating Scale, HDRS) [12],
5. outcomes assessed in the same condition (active stimulation groups only),
6. the same diagnosis of unipolar MDD since both rTMS and DTMS are FDA-approved for unipolar MDD only.

Although the protocol for this review was not published a priori, we have selected the above parameters a priori based on our knowledge of the stimulation designs used in this field and the statistical approach used in our previous meta-analyses of rTMS and DTMS studies [3, 8, 13]. The approach of focusing on a set of homogeneous parameters, rather than on the FDA-approved clinical paradigms or RCTs only, is suited to assess the antidepressant outcomes of the two coils by controlling for as many potential confounding factors as possible. Since we attempt to assess the effects of active stimulation only in studies with any designs (including uncontrolled, open-label studies), our analytical approach is not designed to quantify the clinical efficacy of either coil.

## Methods

### Systematic literature search and study selection

**Fig. 1:**
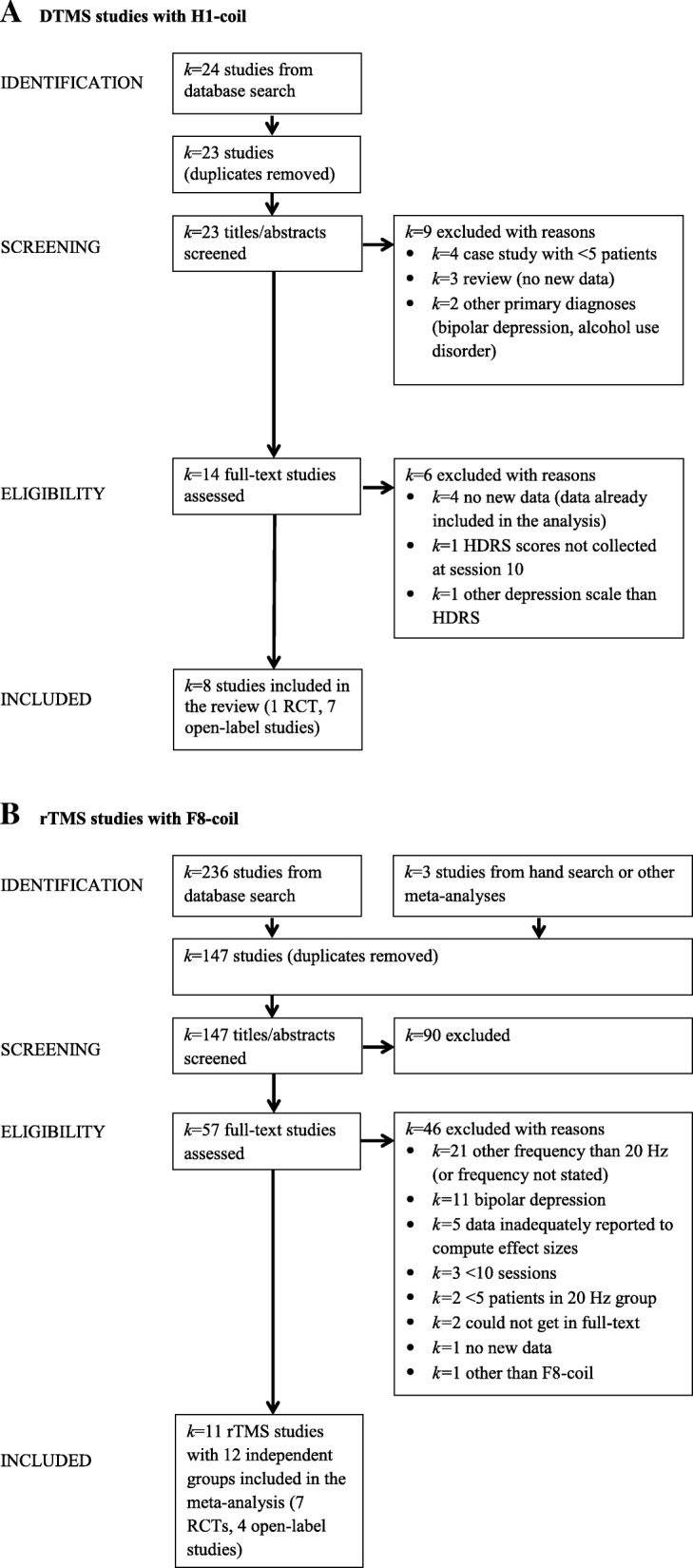
Study selection procedure (PRISMA flowchart). **a**. DTMS studies with H1-coil. **b**. rTMS studies with F8-coil. Note. Abbreviations: DTMS, deep transcranial magnetic stimulation; F8, figure-of-eight coil (rTMS); H1, H1-coil (DTMS); HDRS, Hamilton Depression Rating Scale; *k*, number of studies; RCT, double-blind randomised-controlled trial with an inactive sham group; rTMS, repetitive transcranial magnetic stimulation.

The study was conducted according to the PRISMA guidelines [14]. Details of the systematic literature search are shown in Table 1. Study assessment and selection is summarised on Fig. 1.

**Table 1:** Search strategy

Search	*k* studies	Search terms	Databases (time frame)
DTMS studies with H1-coil	24 (with duplicates)	TI (“deep transcranial magnetic stimulation” OR “deep repetitive transcranial magnetic stimulation” OR deep rTMS OR deepTMS OR deep TMS OR H-coil) AND TI (depress* OR dysthymi* OR MDD)	PsycInfo, Medline (EBSCO); any date – 24.06.2016
rTMS studies with F8-coil	236 (with duplicates)	TI (“repetitive transcranial magnetic stimulation” OR rTMS OR HF-rTMS OR TMS OR “transcranial magnetic stimulation”) AND TI (depress* OR dysthy* OR MDD OR antidepress*) AND TX (“high-frequency” OR “20 Hz”) NOT TI (bilateral OR review OR meta-analysis OR meta-analyses OR case OR bipolar OR “Parkinson’s Disease” OR “posttraumatic stress disorder” OR tinnitus OR “deep transcranial magnetic stimulation” OR “deep TMS” OR H-coil)	PsycInfo, Medline (EBSCO); any date – 24.06.2016

The searches were performed in English (there were no language restrictions or any other limits)

Abbreviations: *DTMS* deep transcranial magnetic stimulation, *F8* figure-of-eight coil (rTMS), *H1* H1-coil (DTMS), *k* number of studies, *MDD* major depressive disorder, *HF-rTMS* high-frequency repetitive transcranial magnetic stimulation, *rTMS* repetitive transcranial magnetic stimulation, *TI* title, *TMS* transcranial magnetic stimulation, *TX* text

A total of *k* = 19 studies (*k* = 8 out of 23 DTMS studies with H1-coil and *k* = 11 out of 147 rTMS studies with F8-coil; Fig. 1) met the following inclusion criteria for the current review:

1. DTMS with H1-coil or rTMS with F8-coil,
2. high-frequency (18–20 Hz) stimulation over the left DLPFC,
3. antidepressant outcomes assessed with any version of HDRS [12],
4. HDRS scores reported at baseline and following 10 daily sessions (the decision to code data at session 10 was made because, as explained above, rTMS with F8-coil and high-frequencies of 18–20 Hz was delivered for 10 days only),
5. at least five patients with unipolar MDD according to DSM-IV,
6. any study designs (RCTs with inactive sham groups or open-label designs to allow for inclusion of all studies on DTMS so far, seven of which were open-label),
7. study published in a peer-reviewed academic journal.

The *k* = 8 DTMS studies with the H1-coil [9, 15–21], all with open-label designs except for the one double-blind RCT with an inactive sham group [9]. The *k* = 11 rTMS studies with the F8-coil and 12 independent subgroups [22–32], including *k* = 7 double-blind RCTs with an inactive sham group [22, 23, 25–27, 31, 32] and *k* = 4 studies with open-label designs [24, 28–30]. Studies were excluded if they 1) did not report new primary data (reviews or previously published data), 2) included patients with bipolar MDD or other primary diagnoses, 3) did not assess depression at session 10, and 4) used other stimulation protocols (frequency of less than 18 Hz and/or less than 10 sessions).

### Data coding

Data regarding study designs and patient characteristics (Table 2), stimulation parameters (Table 3), and clinical outcomes (Table 4) were coded independently by both authors and any inconsistencies were resolved by consensus. All antidepressant outcome data were coded only from groups that received active stimulation. Data from two independent subgroups of patients who received stimulation with different protocols in one rTMS study [31] were included as two primary studies in the current analysis. Therefore, the final meta-analysis included data from 20 independent groups in *k* = 19 studies (*k* = 8 DTMS with H1-coil and *k* = 11 rTMS with F8-coil).

**Table 2:** Study designs and patient characteristics in *k* = 8 DTMS studies with H1-coil and *k =* 11 rTMS studies with F8-coil

Study	Sham-controlled RCT	Sample size baseline	Age all patients baseline (mean ± *SD*)	Female patients baseline	Concurrent antidepressants (% patients baseline)	Dropouts daily stimulation phase before session 10 (number of patients and reasons)	Treatment-resistance definition	Mean onset age (years)	Mean illness duration (years)
DTMS (H1-coil)									
Levkovitz et al., 2009 [15]; Israel^a,b^	No	23	46	48%	0%	3 (all for reasons unrelated to treatment or a good response)	Did not respond to ≥2 antidepressant medications current episode	29	17
Rosenberg et al., 2010 [16]; Israel	No	7	47 ± 12	14%	0%	0	Failed 2 antidepressant trials current episode	33	14
Rosenberg et al., 2010 [17]; Israel	No	6	41 ± 13	67%	50%	0	Failed ≥2 antidepressant courses and ≥ 1 course of ECT	17	24
Isserles et al., 2011 [18]; Israel^c^	No	25	45 ± 13	45%	100%	5 (1 seizure, 1 intolerance, 2 lack of response, 1 high motor threshold)	Failed ≥2 antidepressants	29	17
Harel et al., 2014 [19]; Israel^b^	No	29	41 ± 11	48%	62%	3 (1 safety reasons <1st treatment, 2 non-compliance with study protocol)	Failed ≥1 pharmacological trial or intolerant to 2 antidepressants	24	17
Levkovitz et al., 2015 [9]; multicentre^b,d^	Yes	89	45 ± 12	48%	0%	6 (1 seizure, 3 no improvement, 1 withdrawal of consent, 1 missed > 2 treatment days)	93% failed ≥1 medication	25	20
Rapinesi et al., 2015 [20]; Italy^b^	No	9	54 ± 6	44%	89%	0	Failure to respond to ≥3 adequate doses of ≥2 classes antidepressants	45	9
Rapinesi et al., 2015 [21]; Italy^b,e^	No	12	51 ± 8	42%	100%	0	Unsatisfactory response to ≥1 adequate course of antidepressant treatment current episode	34	17
rTMS (F8-coil)									
George et al., 1997 [22]; USA^b,f^	Yes	7	42 ± 16	86%	some	0	Received 1–21 previous medications	–	–
Berman et al., 2000 [23]; USA^b,d^	Yes	10	45 ± 10	20%	0%	0	Failed ≥1 adequate pharmacologic trial current or previous episodes	20	25
Catafau et al., 2001 [24]; Spain^b^	No	5	50 ± 8	60%	100%	0	HDRS17 score > 18 after ≥6 weeks of treatment	–	–
Garcia-Toro et al., 2001 [25]; Spain^b,d^	Yes	17	52 ± 16	41%	100%	0	Failed 2 antidepressant trials for ≥6 weeks current episode	36	16
Garcia-Toro et al., 2001 [26]; Spain^d^	Yes	11	43 ± 13	55%	100%	3 (no treatment-related adverse reactions)	Failed 1 antidepressant trial	–	–
Boutros et al., 2002 [27]; USA^d^	Yes	12	49 ± 8	33%	100%	1 (worsening of depression)	Failed ≥2 prior medication trials of adequate length and dosages	–	–
Bajbouj et al., 2005 [28]; Germany	No	30	46 ± 12	37%	40%	0	Some non-responders to current treatment with antidepressants	–	–
Yukimasa et al., 2006 [29]; Japan	No	26	53 ± 18	58%	some	0	Failed ≥2 antidepressant trials of adequate duration and dosages	–	–
Luborzewski et al., 2007 [30]; Germany^b^	No	17	46 ± 11	12%	53%	0	Completed 3–19 antidepressant treatment trials	–	–
Bakim et al., 2012 [31]; Turkey^b,g^	Yes; 80%	12	39 ± 10	83%	100%	0	No response to adequate courses (≥6 weeks) of ≥2 different classes of antidepressants at optimal doses	36	3
Bakim et al., 2012 [31]; Turkey^b,g^	Yes; 110%	11	43 ± 8	91%	100%	0	No response to adequate courses (≥6 weeks) of ≥2 different classes of antidepressants at optimal doses	40	3
Chen et al., 2013 [32]; Taiwan^d^	Yes	10	44 ± 4	70%	100%	0	No response to 2 different antidepressants administered for 6 weeks each	–	–

All studies include patients with MDD according to DSM-IV. Mean onset age = mean age – mean illness duration. Mean illness duration = mean age – mean onset age. ^a^Data from H1–120% group (other groups were stimulated with different H-coil types). ^b^Data from unipolar MDD patients. ^c^Data from the control group ‘No cognitive-emotional reactivation’ (other groups received cognitive-emotional priming prior to DTMS). ^d^Data from the active stimulation group. ^e^Data from MDD group without alcohol use disorders. ^f^Data from the active rTMS group at week 2, phase 1 (before cross-over). ^g^Data from two independent groups who received active rTMS with different resting motor thresholds: 80% or 110%

Abbreviations: *DTMS* deep transcranial magnetic stimulation, *ECT* electroconvulsive therapy, *F8* figure-of-eight coil (rTMS), *H1* H1-coil (DTMS), *HDRS* Hamilton Depression Rating Scale, *k* number of studies, *MDD* major depressive disorder, *RCT* double-blind randomised-controlled trial with an inactive sham group, *rTMS* repetitive transcranial magnetic stimulation, *SD* standard deviation

**Table 3:** Active stimulation parameters in *k* = 8 DTMS studies with H1-coil and *k =* 11 rTMS studies with F8-coil

Study	PFC location	Location definition	Frequency (Hz)	Intensity (% MT)	Coil type	Total stimuli	Stimuli/session	Trains/session	Inter-train interval (s)	No. of sessions
DTMS (H1-coil)										
Levkovitz et al., 2009 [15]	L	5.5 cm	20	120	H1	16,800	1680	42	20	10
Rosenberg et al., 2010 [16]	L	5.5 cm	20	120	H1	16,800	1680	42	20	10
Rosenberg et al., 2010 [17]	L	5.5 cm	20	120	H1	16,800	1680	42	20	10
Isserles et al., 2011 [18]	L	5.5 cm	20	120	H1	16,800	1680	42	20	10
Harel et al., 2014 [19]	L	6 cm	20	120	H1	16,800	1680	42	20	10
Levkovitz et al., 2015 [9]	L	6 cm	18	120	H1	19,800	1980	55	20	10
Rapinesi et al., 2015 [20]	L	5.5 cm	18	120	H1	19,800	1980	55	20	10
Rapinesi et al., 2015 [21]	L	5.5 cm	18	120	H1	19,800	1980	55	20	10
rTMS (F8-coil)										
George et al., 1997 [22]	L	5 cm	20	80	F8	8000	800	–	58	10
Berman et al., 2000 [23]	L	5 cm	20	80	F8	–	–	20	58	10
Catafau et al., 2001 [24]	L	5 cm	20	90	F8	12,000	1200	30	30	10
Garcia-Toro et al., 2001 [25]	L	5 cm	20	90	F8	–	–	30	30	10
Garcia-Toro et al., 2001 [26]	L	5 cm	20	90	F8	12,000	1200	30	30	10
Boutros et al., 2002 [27]	L	5 cm	20	80	F8	8000	800	20	58	10
Bajbouj et al., 2005 [28]	L	5 cm	20	100	F8	20,000	2000	50	–	10
Yukimasa et al., 2006 [29]	L	5 cm	20	80	F8	8000	800	–	–	10
Luborzewski et al., 2007 [30]	L	5 cm	20	100	F8	20,000	2000	50	–	10
Bakim et al., 2012 [31] 80%	L	5 cm	20	80	F8	8000	800	20	60	10
Bakim et al., 2012 [31] 110%	L	5 cm	20	110	F8	8000	800	20	60	10
Chen et al., 2013 [32]	L	5 cm	20	90	F8	–	–	20	10	10

DTMS was applied in 20 daily sessions in all studies. Since rTMS was applied in 10 sessions in most studies, data at 10 sessions were coded in all DTMS and rTMS studies. For the definition of location, ‘5.5 cm’ refers to 5.5 cm away from the motor ‘hot-spot’. Abbreviations: *DTMS* deep transcranial magnetic stimulation, *F8* figure-of-eight coil (rTMS), *H1* H1-coil (DTMS), *k* number of studies, *L* left PFC, *MT* resting motor threshold, *PFC* prefrontal cortex, *rTMS* repetitive transcranial magnetic stimulation

**Table 4:** Antidepressant outcomes in *k* = 8 DTMS studies with H1-coil and *k =* 11 rTMS studies with F8-coil

Study	Response rate (session 10)	Remission definition	Remission rate (session 10)	Scale	Baseline daily phase; Mean ± *SD* (*n*)	Session 10 daily phase; Mean ± *SD* (*n*)
DTMS (H1-coil)						
Levkovitz et al., 2009 [15]^a,b^	45% (9/20)	HDRS≤10	20% (4/20)	HDRS24	31 ± 5 (20)	19 ± 8 (20)
Rosenberg et al., 2010 [16]	29% (2/7)	HDRS≤10	14% (1/7)	HDRS24	27 ± 4 (7)	18 ± 6 (7)
Rosenberg et al., 2010 [17]	50% (3/6)	HDRS≤10	17% (1/6)	HDRS24	31 ± 4 (6)	17 ± 7 (6)
Isserles et al., 2011 [18]^c^	–	–	–	HDRS24	29 ± 6 (20)	16 ± 4 (20) completers
Harel et al., 2014 [19]^b^	–	–	–	HDRS21	23 ± 4 (29)	17 ± 3 (26) completers
Levkovitz et al., 2015 [9]^b,d^	15% (13/89)	HDRS≤10	7% (6/89)	HDRS21	24 ± 4 (89)	18 ± 6 (83)
Rapinesi et al., 2015 [20]^b^	0%	HDRS≤10	11% (1/9)	HDRS21	24 ± 3 (9)	15 ± 3 (9)
Rapinesi et al., 2015 [21]^b,e^	0%	HDRS≤7	0%	HDRS17	27 ± 6 (12)	22 ± 5 (12)
rTMS (F8-coil)						
George et al., 1997 [22]^b,f^	14% (1/7)	HDRS≤10	14% (1/7)	HDRS21	30 ± 4 (7)	23 ± 9 (7)
Berman et al., 2000 [23]^b,d^	10% (1/10)	HDRS≤10	10% (1/10)	HDRS25	37 ± 10 (10)	25 ± 9 (10)
Catafau et al., 2001 [24]^b^	40% (2/5)	HDRS≤7	0%	HDRS17	22 ± 4 (5)	19 ± 9 (5)
Garcia-Toro et al., 2001 [25]^b,d^	–	–	–	HDRS21	27 ± 7 (17)	20 ± 6 (17)
Garcia-Toro et al., 2001 [26]^d^	36% (4/11)	–	–	HDRS21	26 ± 6 (11)	16 ± 8 (11) completers
Boutros et al., 2002 [27]^d^	25% (3/12)	HDRS≤10	8% (1/12)	HDRS25	41 ± 10 (12)	29 ± 14 (12) LOCF
Bajbouj et al., 2005 [28]	33% (10/30)	–	–	HDRS24	26 ± 7 (30)	18 ± 9 (30)
Yukimasa et al., 2006 [29]	19% (5/26)	HDRS≤7	27% (7/26)	HDRS17	21 ± 5 (26)	16 ± 7 (26)
Luborzewski et al., 2007 [30]^b^	35% (6/17)	HDRS≤10	29% (5/17)	HDRS28	25 ± 7 (17)	19 ± 11 (17)
Bakim et al., 2012 [31] 80%^b,g^	–	–	–	HDRS17	23 ± 4 (12)	16 ± 6 (12)
Bakim et al., 2012 [31] 110%^b,g^	–	–	–	HDRS17	24 ± 3 (11)	17 ± 5 (11)
Chen et al., 2013 [32]^d^	70% (7/10)	–	–	HDRS17	24 ± 2 (10)	10 ± 2 (10)

Remission was defined as HDRS≤7 for HDRS-17 and HDRS≤10 for any other version of HDRS

^a^Data from H1–120% group (other groups were stimulated with different H-coil types). ^b^Data from unipolar MDD patients. ^c^Data from the control group ‘No cognitive-emotional reactivation’ (other groups received cognitive-emotional priming prior to DTMS). ^d^Data from the active stimulation group. ^e^Data from MDD group without alcohol use disorders. ^f^Data from the active rTMS group at week 2, phase 1 (before cross-over). ^g^Data from two independent groups who received active rTMS with different resting motor thresholds: 80% or 110%

Abbreviations: *DTMS* deep transcranial magnetic stimulation, *F8* figure-of-eight coil (rTMS), *H1* H1-coil (DTMS), *HDRS* Hamilton Depression Rating Scale, *k* number of studies, *LOCF* last observation carried forward, *n* sample size, *rTMS* repetitive transcranial magnetic stimulation, *SD* standard deviation

If patients dropped out before session 10, the last observation carried forward (LOCF) or intention-to-treat (ITT) approaches were used to code data (unless data for completers only were reported in a study).

### Outcome measures

The current review focuses on the following outcomes:

1. primary outcome: depression severity defined as a standardised change in HDRS depression scores at session 10 relative to baseline,
2. secondary outcomes:
  - response rates defined as at least 50% reduction in HDRS score from baseline,
  - remission rates defined as scores of HDRS≤7 for HDRS-17 and HDRS≤10 for any other version of HDRS (these cut-off values were used to standardise the results among studies).

### Data analysis

We use the same approach to meta-analysis as described elsewhere [8]. The formulae for the effect sizes used in the current analysis are shown in the supplementary materials to our earlier meta-analysis [8].

Data from primary studies were expressed as effect sizes. The primary outcomes (depression severity scores) were expressed as Hedges’ *g*, standardised paired differences in means (baseline – session 10) adjusted for the sample sizes *n* [33]. There are three advantages of using Hedges’ *g*. First, Hedges’ *g* is a mean (paired) difference score meaning that the average depression severity at session 10 is corrected for baseline. Second, Hedges’ *g* is a standardised effect size meaning that the variability of scores (standard deviations) at both points in time (baseline and at session 10) is included in the computation. Third, Hedges’ *g* is adjusted for the sample size in each study to reduce the inflation of effect sizes in studies with small *n* [33]. Hedges’ *g* was interpreted using the same criteria as for Cohen’s *d* (.20–.49 small, .50–.79 moderate, ≥.80 large effect) [33]. Given our calculation, positive values of *g* indicate a reduction in depression severity after treatment relative to baseline. The secondary outcomes (response and remission rates) were expressed as event rates (number of responders or remitters out of the total sample per study).

All analyses were carried out with Comprehensive Meta-Analysis 3.0 software (Biostat, USA). The effect sizes were weighted using the inverse-variance method (the inverse of the sum of the within- and between-study variance) [33]. The weighted effects were pooled using a random-effects model of meta-analysis. The random-effects model was chosen because it was assumed that a random sample of all studies on the topic was included in the analysis, that the true effect sizes would vary due to methodological heterogeneity among studies, and that is it possible to generalise the findings beyond the studies included in this meta-analysis [33]. Heterogeneity among study effect sizes was measured with an *I*^*2*^ index based on a *Q* statistic [33]. The *I*^*2*^ index was interpreted as follows: *I*^*2*^ ≤ 25% reflects little, 50% moderate, and ≥ 75% high heterogeneity [33].

In the first part of the analysis all effect sizes were pooled into the overall weighted effects to assess the antidepressant outcomes in studies with either coil (F8-coil and H1-coil). Univariate random-effects meta-regressions were used to test if the weighted effects could be predicted using patient characteristics (demographic and clinical) and stimulation parameters. Univariate approach was used because data from 15 cases (15 studies) are required per predictor in a standard linear regression analysis. Mixed-effects subgroup analyses were also computed to test for differences in the pooled effects between subgroups of studies with either coil based on study design (RCT vs. open-label designs) or therapy type (monotherapy vs. add-on to concurrent antidepressants). The mixed-effects analysis consisted of the random-effects model that was used to pool the effect sizes within each subgroup of studies and the fixed-effect model that was used to compute a between-groups *Q* statistic to test for the difference between two pooled effects.

In the second part of the analysis the pooled effect sizes were compared between subgroups of studies using F8-coils vs. H1-coils according to the mixed-effects model described above. Sensitivity analyses were also carried out to find out if any differences in the pooled effects in studies with F8-coils vs. H1-coils were due to study design or therapy type.

Publication bias was assessed using funnel plots, and Rosenthal’s and Orwin’s Fail-Safe *Ns* [33]. Funnel plots show the distribution of study effect sizes vs. variability (expressed as the standard error of the mean, *SEM*) around the pooled effect size of all studies in the analysis. Duval and Tweedie’s trim-and-fill analysis was applied to assesses the mathematical symmetry of the plot. It was assumed that a lack of symmetry is attributable to the publication bias. In such cases, the pooled effect sizes were adjusted for theoretically missing effect sizes necessary to make the plots symmetrical. If the adjustment changes the interpretation of the pooled effect size then the impact of publication bias is severe and invalidates the results of meta-analysis [33]. Rosenthal’s and Orwin’s Fail-Safe *Ns* are the number of studies with small effect sizes theoretically missing from the analysis that could reduce the pooled effect sizes to zero (Rosenthal’s Fail-Safe *N)* or to less than a trivial effect (Orwin’s Fail-Safe *N*) [33]. The criteria for ‘trivial’ effect sizes and estimated mean effect sizes in missing studies necessary to compute Orwin’s Fail-Safe *Ns* are shown in the supplementary materials for each analysis separately.

## Results

### Study designs and patient characteristics

Patient characteristics were similar in all studies irrespective of the coil (Table 2). A total of *n* = 368 patients (*n* = 168 in *k* = 11 rTMS studies with F8-coil and *n* = 200 in *k* = 8 DTMS studies with H1-coil), mostly with treatment-resistant MDD were included in *k* = 19 studies with 20 independent subgroups (Table 2). Treatment-resistance was most often defined as a failure to respond to at least two courses of antidepressants of adequate length and dosage. The mean onset age of depression was 20–40 years (F8-coil studies) or 17–45 years (H1-coil studies), and the mean duration of illness was 3–25 years (F8-coil studies) or 9–24 years (H1-coil studies). Most studies applied rTMS or DTMS as add-on therapies to stable doses of antidepressants (10/11, F8-coil studies and 5/8, H1-coil studies, respectively). The patients were on average middle aged (39–53 years, F8-coil studies; 41–54 years, H1-coil studies) and 12–91% (F8-coil studies) or 14–67% (H1-coil studies) were female.

The main systematic difference between rTMS with F8-coil and DTMS with H1-coil was the study design. Specifically, most rTMS studies with F8-coil were sham-controlled RCTs (7/11 studies) while most DTMS studies with H1-coil utilised open-label designs (7/8 studies).

### Stimulation parameters

All studies irrespective of the coil utilised high-frequencies (18–20 Hz) and measured depression outcomes after 10 daily treatment sessions (although all DTMS protocols continued for 20 daily sessions while most rTMS protocols with such a high frequency were applied for 10 days only); Table 3. The left DLPFC was targeted in all rTMS studies and presumably also in all DTMS studies although, unlike F8-coil, the H1-coil is not focal and the helmet-like structure of the coil means that the entire brain surface is stimulated to some degree. DLPFC was localised using 5 cm (F8-coil studies) or 5.5–6 cm (H1-coil studies) distance from the motor hot-spot.

There were three systematic differences in the stimulation parameters between the rTMS studies with F8-coil and the DTMS studies with H1-coil. First, stimulation intensities were lower in the rTMS studies (80–110% MT) than in the DTMS studies (120% MT). Second, the number of stimuli was on average lower in the rTMS studies (800–2000 per session) than in the DTMS studies (1680–1980 per session). Consequently, the number of trains per session was mostly lower in the rTMS studies (20–50) than in the DTMS studies (42–55). Third, the inter-train-intervals were longer in most of the rTMS studies (30–60 s) than in the DTMS studies (20 s).

### Acceptability

In general, both methods were well-tolerated with low dropout rates before 10 sessions of treatment (Table 2). Although most reasons for dropping out were non-treatment related and no seizures were reported in the rTMS studies with F8-coil, one out of the four patients who dropped out reported worsening of depression (Table 2). Out of 17 patients who dropped out from the DTMS studies with H1-coil, two experienced a seizure, while another patient did not tolerate the treatment (Table 2).

### Antidepressant outcomes: all studies (rTMS with F8-coil and DTMS with H1-coil)

The antidepressant outcomes of stimulation with either coil in all studies (rTMS with F8-coil and DTMS with H1-coil) are shown in Table 5.

**Table 5:** Meta-analysis of antidepressant outcomes in all studies with either coil (*k* = 19 with 20 independent groups)

Random-effects analyses	Primary outcome (depression severity); Hedges’ *g (95% CI*)	Secondary outcome (response rates); responders/total *n (95% CI*)	Secondary outcome (remission rates); remitters/total *n (95% CI*)
Pooled weighted effect			
Mean (95% *CI*)*; k; n*	1.20 (.96–1.44); *k* = 20; *n* = 351	29% (20–39%); *k* = 15; *n* = 271 (66/271)	15% (10–22%); *k* = 12; *n* = 220 (28/220)
Heterogeneity statistics	*Q* (*df* 19) = 49.12; *p* < .001*; *I*^*2*^ *=* 61%	*Q* (*df* 14) = 27.26; *p* = .018*; *I*^*2*^ *=* 49%	*Q* (*df* 11) = 12.19; *p* = .350; *I*^*2*^ *=* 10%
Publication bias analysis			
Fail-Safe *N* Rosenthal/Orwin	*N*_*Rosenthal*_ = 1372; *N*_*Orwin*_ = 182	*N*_*Rosenthal*_ = 124; *N*_*Orwin*_ = 25	*N*_*Rosenthal*_ = 187; *N*_*Orwin*_ = 8
Funnel plot symmetric?	*No: k* = 4 missing with small effect sizes	*No: k* = 2 missing with large response rates	*No: k* = 6 missing with large remission rates
Mean effect (95% *CI*) adjusted for missing studies	1.05 (.78–1.32)	31% (22–42%)	23% (14–34%)
Subgroup analysis			
Study design			
RCT	1.17 (.86–1.48); *k* = 9; *n =* 173	26% (13–47%); *k* = 6; *n* = 139 (29/139)	8% (4–14%); *k* = 4; *n* = 118 (9/118)
Open-label	1.24 (.87–1.61); *k* = 11; *n =* 178	32% (23–42%); *k* = 9; *n* = 132 (37/132)	21% (14–31%); *k* = 8; *n* = 102 (19/102)
RCT vs. open-label	*Q (df* 1) = .14, *p* = .712	*Q (df* 1) = 3.03, *p* = .082	*Q (df* 1) = 7.31, *p* = .007*
Therapy			
Add-on	1.20 (.89–1.50); *k* = 16; *n =* 231	31% (22–43%); *k* = 11; *n* = 145 (41/145)	20% (13–31%); *k* = 8; *n* = 94 (16/94)
Monotherapy	1.21 (.98–1.44); *k* = 4; *n =* 120	23% (10–45%); *k* = 4; *n* = 126 (25/126)	11% (6–19%); *k* = 4; *n* = 126 (12/126)
Add-on vs. monotherapy	*Q (df* 1) = 1.15, *p* = .283	*Q (df* 1) = 3.09, *p* = .079	*Q (df* 1) = 3.63, *p* = .057
Meta-regression predictors			
Mean age	b = −.05; p = .019*; R^*2*^ *= 45%; k* = 18	*b = −.11; p = .123; R*^*2*^ *= 2%; k* = 15	*b = .06; p = .337; R*^*2*^ *= 23%; k* = 12
% female	*b < .01; p = .642; R*^*2*^ *= 0%; k* = 19	*b < .01; p = .491; R*^*2*^ *= 0%; k* = 15	*b < −.01; p = .696; R*^*2*^ *= 0%; k* = 12
Mean illness duration	*b < −.01; p = .681; R*^*2*^ *= 0%; k* = 12	*b = .05; p = .629; R*^*2*^ *= 0%; k* = 7	*b = −.04; p = .620; R*^*2*^ *= 0%; k* = 7
Mean onset age	*b < .01; p = .913; R*^*2*^ *= 0%; k* = 12	*b = −.06; p = .327; R*^*2*^ *= 0%; k* = 7	*b = .01; p = .811; R*^*2*^ *= 0%; k* = 7
Stimuli/session	*b < .01; p = .415; R*^*2*^ *= 0%; k* = 17	*b < .01; p = .964; R*^*2*^ *= 0%; k* = 13	*b < −.01; p = .313; R*^*2*^ *= 25%; k* = 11
Trains/session	*b < .01; p = .804; R*^*2*^ *= 0%; k* = 17	*b = −.03; p = .132; R*^*2*^ *= 32%; k* = 13	*b < −.01; p = .858; R*^*2*^ *= 0%; k* = 10
Intensity (%MT)	b = .02; p = .007*; R^*2*^ *= 37%; k* = 19	*b < −.01; p = .897; R*^*2*^ *= 0%; k* = 15	*b = −.02; p = .087; R*^*2*^ *= 100%; k* = 12
Inter-train interval (s)	*b = −.01; p = .102; R*^*2*^ *= 0%; k* = 17	*b = −.02; p = .234; R*^*2*^ *= 0%; k* = 12	*b < .01; p = .996; R*^*2*^ *= 0%; k* = 10

The overall analyses and meta-regressions were conducted using the random-effects model. The subgroup analyses were conducted using the mixed-effects model. The *Q*-statistic has two functions: 1) a test for heterogeneity among the effect sizes, 2) a test for differences in effect sizes in subgroup analyses

Abbreviations: *b* unstandardised weighted regression coefficient, *CI* 95% confidence interval, *df* degrees of freedom, *Hedges’ g (effect size)* standardised paired difference in means corrected for the sample size, *k* number of studies, *n* sample size, *%MT* percent of the resting motor threshold, *RCT* double-blind randomised-controlled trial with an inactive sham group

*p_*two-tailed*_ < .05

#### Primary outcome (depression severity)

**Fig. 2:**
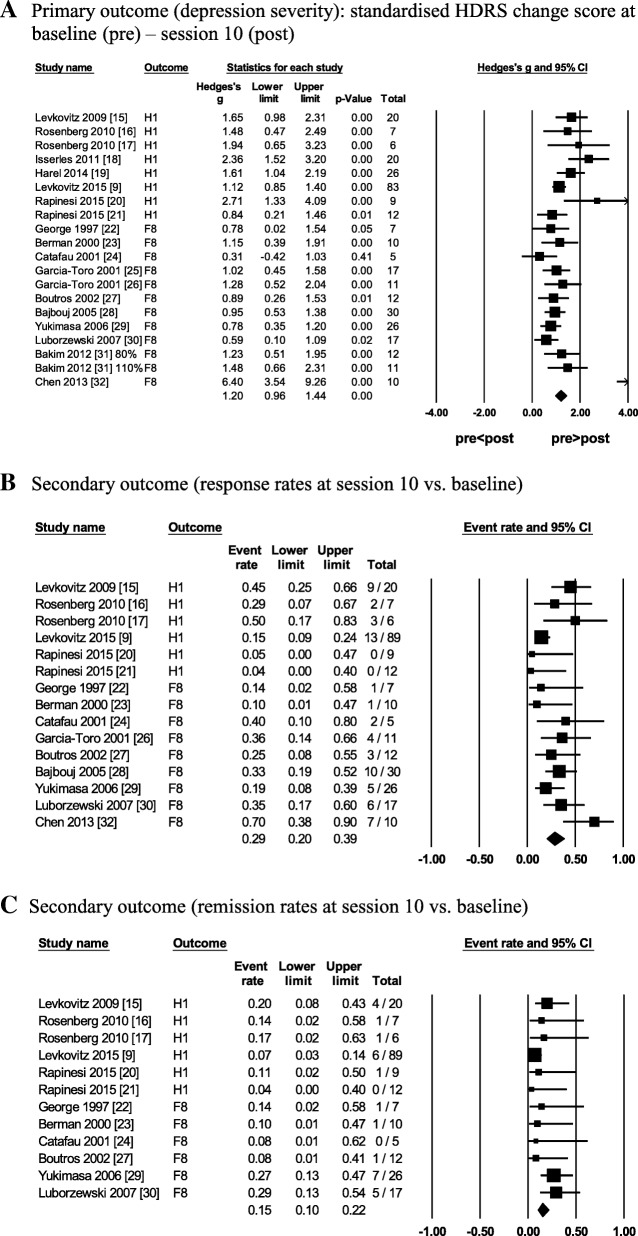
Antidepressant outcomes in all studies with either coil (F8-coil and H1-coil). **a**. Primary outcome (depression severity): standardised HDRS change score at baseline (pre) – session 10 (post). **b**. Secondary outcome (response rates at session 10 vs. baseline). c. Secondary outcome (remission rates at session 10 vs. baseline). Note. Figures **a**-**c** are forest plots of random-effects meta-analyses of the antidepressant outcomes in all studies with either coil (F8-coil and H1-coil). Each forest plot shows the following information: the antidepressant outcomes expressed as effect sizes in each study (Hedges’ *g* or event rates depicted as squares), the estimated 95% *CI* of each effect size (reported in the *Lower limit* and the *Upper limit* columns and shown as horizontal lines), the study weights (depicted as squares with different sizes- the larger the square, the higher the study weight), the study sample sizes (reported in the *Total* columns), and the pooled mean weighted effect sizes with 95% *CI* of all studies (depicted as diamonds- the length of the diamond corresponds to the 95% *CI* of the pooled effect). Abbreviations: *CI*, 95% confidence interval; DTMS, deep transcranial magnetic stimulation; F8, figure-of-eight coil (rTMS); H1, H1-coil (DTMS); HDRS, Hamilton depression rating scale; Hedges’ *g* (effect size), standardised paired difference in means corrected for the sample size; rTMS, repetitive transcranial magnetic stimulation; Total, sample size per study

**Fig. 3:**
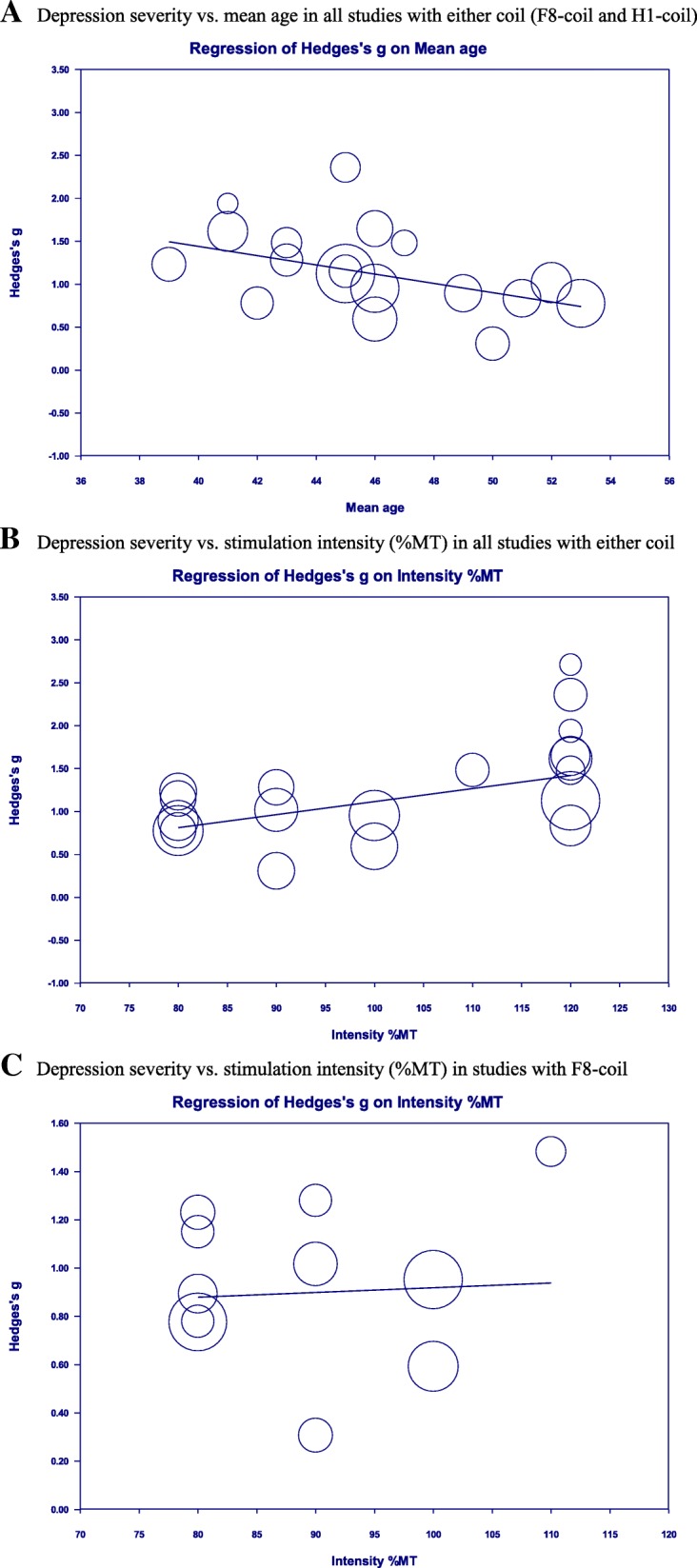
Relationships between primary outcome (depression severity), mean age, and stimulation intensity. **a**. Depression severity vs. mean age in all studies with either coil (F8-coil and H1-coil). **b**. Depression severity vs. stimulation intensity (%MT) in all studies with either coil. **c**. Depression severity vs. stimulation intensity (%MT) in studies with F8-coil. Note. Figures **a**-**c** are scatterplots of random-effects meta-regressions. All plots show the relationships between depression severity expressed as weighted effect sizes in each study (Hedges’ *g* depicted as circles- the larger the circle, the higher the study weight) on the Y-axes and predictors on the X-axes (mean age of all patients per study or stimulation intensity per study). Outlier studies were excluded from the analyses. Abbreviations: DTMS, deep transcranial magnetic stimulation; F8, figure-of-eight coil (rTMS); H1, H1-coil (DTMS); Hedges’ *g* (effect size), standardised paired difference in means corrected for the sample size; %MT, percent of the resting motor threshold; rTMS, repetitive transcranial magnetic stimulation

There was a large, significant reduction in depression severity after 10 sessions relative to baseline in all studies (*g* = 1.20; Table 5; Fig. 2a). A moderate heterogeneity among the effect sizes (*I*^*2*^ = 61%) was in part due to one outlier study [32] (Additional file 1: Figure S1a). Although removing the study reduced the heterogeneity to 50%, the interpretation of the results above did not change and thus the study was kept in all analyses (Additional file 1: Figure S1b). Depression severity did not depend on the study design (RCT vs. open-label; Additional file 1: Figure S2a) or the therapy type (add-on to antidepressants vs. monotherapy; Additional file 1: Figure S2b) according to the subgroup analyses (Table 5). However, the reduction in depression severity was significantly predicted by two factors: the mean patient age and the stimulation intensity (%MT) per study in the meta-regression analyses (Table 5). Specifically, the reduction in depression severity was greater in studies with younger patients (Fig. 3a) and in studies with higher stimulation intensity (Fig. 3b). The latter relationship was likely due to the coil-type rather than the stimulation intensity because all the DTMS studies with H1-coil used the highest intensity (120% MT) and removing these studies from the analysis also removed the significance of the meta-regression (i.e. the stimulation intensity did not predict depression severity in rTMS studies with F8-coil alone; Fig. 3c). There was little evidence for publication bias in this analysis (Table 5; Additional file 1: Figure S3).

#### Secondary outcome (response rates)

Twelve studies reported response and remission rates (*k* = 6 with F8-coil and *k* = 6 with H1-coil). A total of 66 out of 271 patients responded to stimulation with either coil (F8-coil or H1-coil) after 10 sessions (pooled response rate of 29%; Table 5; Fig. 2b). There was a moderate heterogeneity among the effect sizes (*I*^*2*^ = 49%). The response rates did not depend on the study design (RCT vs. open-label) or the therapy type (add-on to antidepressants vs. monotherapy) according to the subgroup analyses and were not predicted by any factors, including demographics, the clinical characteristics of patients, or the stimulation parameters in meta-regressions (Table 5). However, trends in the data indicate that the pooled response rates tended to be higher in studies with the open-label designs (32%) relative to RCTs (26%; *p* = .082; Additional file 1: Figure S4a) and in studies with patients on concurrent antidepressants (31%) relative to the monotherapy (23%; *p* = .079; Table 5; Additional file 1: Figure S4b). There was little evidence for publication bias in this analysis (Table 5; Additional file 1: Figure S5).

#### Secondary outcome (remission rates)

A total of 28 out of 220 patients remitted after 10 sessions of stimulation with either coil (F8-coil or H1-coil) with a pooled remission rate of 15% (Table 5; Fig. 2c). There was a low heterogeneity among the effect sizes (*I*^*2*^ = 10%). The pooled remission rates were significantly higher in studies with the open-label designs (21%) relative to RCTs (8%; *p* = .007; Additional file 1: Figure S6a) and tended to be higher in studies with patients on concurrent antidepressants (20%) relative to the monotherapy (11%; *p* = .057; Additional file 1: Figure S6b). Remission rates were not predicted by any factors, including demographics, the clinical characteristics of patients, or the stimulation parameters in meta-regressions but tended to be lower in studies with the higher stimulation intensity (*p* = .087; Additional file 1: Figure S7). There was little evidence for publication bias in this analysis (Table 5; Additional file 1: Figure S8).

### Antidepressant outcomes: rTMS with F8-coil vs. DTMS with H1-coil

The comparisons in antidepressant outcomes between rTMS studies with F8-coil and DTMS studies with H1-coil are shown in Table 6.

**Table 6:** Meta-analysis of antidepressant outcomes in DTMS studies with H1-coil vs. rTMS studies with F8-coil

Mixed-effects analyses	Primary outcome (depression severity); Hedges’ *g (95% CI*)	Secondary outcome (response rates); responders/total *n (95% CI*)	Secondary outcome (remission rates); remitters/total *n (95% CI*)
Pooled weighted effects			
Mean (95% *CI*); *k; n*			
DTMS (H1-coil)	1.55 (1.17–1.94); *k* = 8; *n* = 183	24% (11–44%); *k* = 6; *n* = 143 (27/143)	10% (6–17%); *k* = 6; *n* = 143 (13/143)
rTMS (F8-coil)	.97 (.70–1.25); *k* = 12; *n* = 168	31% (22–43%); *k* = 9; *n* = 128 (39/128)	22% (14–33%); *k* = 6; *n* = 77 (15/77)
H1-coil vs. F8-coil	*Q* (*df* 1) = 9.32, *p* = .002*	*Q* (*df* 1) = 2.46, *p* = .116	*Q* (*df* 1) = 4.46, *p* = .035*
Heterogeneity statistics			
DTMS (H1-coil)	*Q* (*df* 7) = 16.89, *p* = .018*; *I*^*2*^ *=* 58%	*Q* (*df* 5) = 14.13, *p* = .015*; *I*^*2*^ *=* 65%	*Q* (*df* 5) = 4.05, *p* = .543; *I*^*2*^ *=* 0%
rTMS (F8-coil)	*Q* (*df* 11) = 22.91, *p* = .018*; *I*^*2*^ *=* 52%	*Q* (*df* 8) = 10.67, *p* = .221; *I*^*2*^ *=* 25%	*Q* (*df* 5) = 3.68, *p* = .596; *I*^*2*^ *=* 0%
Sensitivity analyses			
Open-label studies only			
DTMS (H1-coil)	1.67 (1.24–2.11); *k* = 7; *n =* 100	29% (12–53%); *k* = 5; *n =* 54 (14/54)	15% (8–28%); *k* = 5; *n* = 54 (7/54)
rTMS (F8-coil)	.74 (.50–.98); *k* = 4; *n =* 78	30% (21–41%); *k* = 4; *n =* 78 (23/78)	26% (16–41%); *k* = 3; *n* = 48 (12/48)
H1-coil vs. F8-coil	*Q (df* 1) = 19.14, *p* < .001*	*Q (df* 1) = .34, *p* = .559	*Q* (*df* 1) = 1.76, *p* = .184
Add-on studies only			
DTMS (H1-coil)	1.77 (1.12–2.42); *k* = 5; *n =* 73	14% (2–62%); *k* = 3; *n* = 27 (3/27)	10% (3–30%); *k* = 3; *n* = 27 (2/27)
rTMS (F8-coil)	.96 (.67–1.26); *k* = 11; *n =* 158	33% (24–44%); *k* = 8; *n* = 118 (38/118)	23% (14–35%); *k* = 5; *n* = 67 (14/67)
H1-coil vs. F8-coil	*Q (df* 1) = 11.46, *p* = .001*	*Q* (*df* 1) = .52, *p* = .473	*Q* (*df* 1) = 1.66, *p* = .197

Abbreviations: *CI* 95% confidence interval, *df* degrees of freedom, *DTMS* deep transcranial magnetic stimulation, *F8* figure-of-eight coil (rTMS), *H1* H1-coil (DTMS), *Hedges’ g (effect size)* standardised paired difference in means corrected for the sample size, *k* number of studies, *n* sample size, *rTMS* repetitive transcranial magnetic stimulation

*p_*two-tailed*_ < .05

#### Primary outcome (depression severity)

**Fig. 4:**
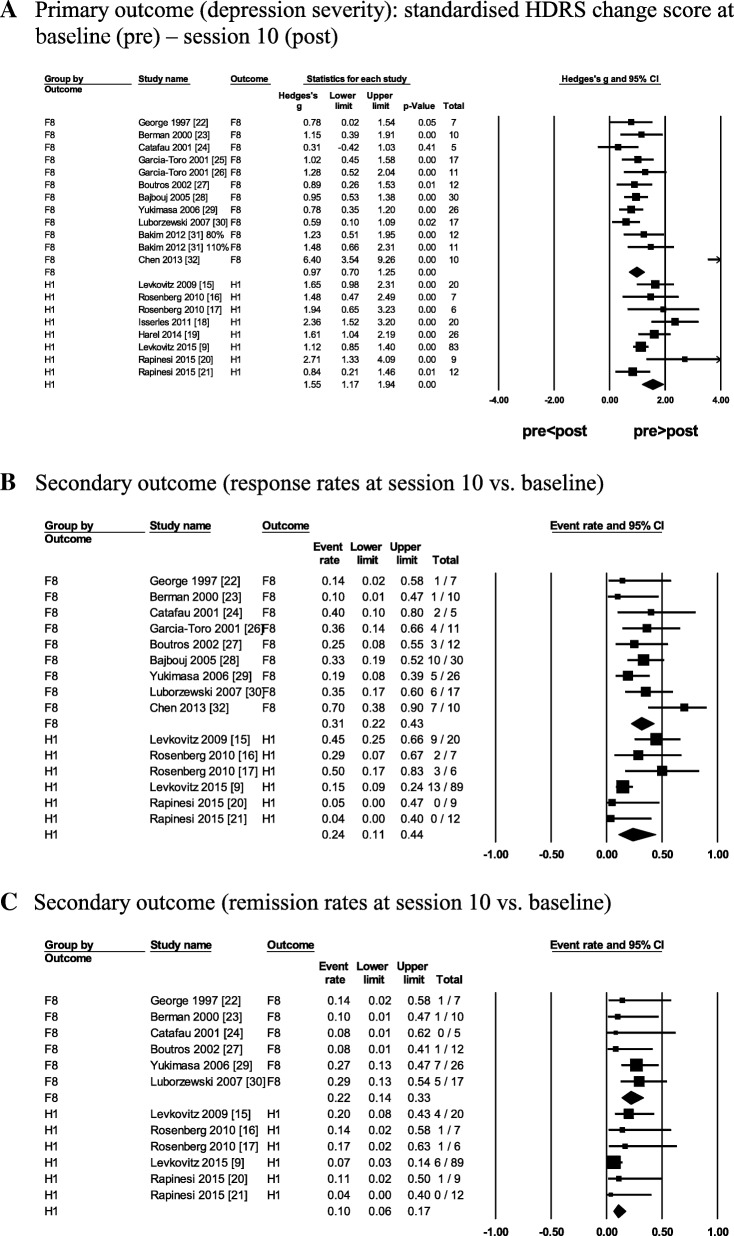
Antidepressant outcomes in DTMS studies with H1-coil vs. rTMS studies with F8-coil. **a**. Primary outcome (depression severity): standardised HDRS change score at baseline (pre) – session 10 (post). **b**. Secondary outcome (response rates at session 10 vs. baseline). **c**. Secondary outcome (remission rates at session 10 vs. baseline). Note. Figures **a**-**c** are forest plots of mixed-effects meta-analyses comparing the antidepressant outcomes in studies with F8-coil vs. H1-coil. In contrast to Fig. 2, each forest plot shows two diamonds corresponding to the pooled mean weighted effects of studies with F8-coil (the upper diamonds) vs. studies with H1-coil (the lower diamonds). Abbreviations: *CI*, 95% confidence interval; DTMS, deep transcranial magnetic stimulation; F8, figure-of-eight coil (rTMS); H1, H1-coil (DTMS); HDRS, Hamilton depression rating scale; Hedges’ *g* (effect size), standardised paired difference in means corrected for the sample size; rTMS, repetitive transcranial magnetic stimulation; Total, sample size per study

The reduction in depression severity had a significantly (*p* = .002) larger effect size after 10 sessions of DTMS with H1-coil (pooled *g* = 1.55) relative to rTMS with F8-coil (pooled *g* = .97; Table 6; Fig. 4a). This result did not depend on the study design or the therapy type according to the subgroup analyses. Specifically, the reduction in depression severity remained significantly larger after DTMS with H1-coil relative to rTMS with F8-coil in studies with open-label designs (*g* = 1.67 vs. *g* = .74, respectively; *p* < .001) and in studies with patients on concurrent antidepressants (*g* = 1.77 vs. *g* = .96, respectively; *p* = .001; Table 6; Additional file 1: Figure S9).

#### Secondary outcome (response rates)

The pooled response rates tended to be similar and did not differ statistically after 10 sessions of DTMS with H1-coil relative to rTMS with F8-coil (Table 6; Fig. 4b). The pooled response rates also did not differ statistically after rTMS relative to DTMS in studies with the open-label designs only and in studies with patients on concurrent antidepressants (although a non-significant trend towards higher response rates was seen after rTMS vs. DTMS; Table 6; Additional file 1: Figure S10).

#### Secondary outcome (remission rates)

The pooled remission rates were significantly higher after 10 sessions of rTMS with F8-coil (22%) relative to DTMS with H1-coil (10%; *p* = .035; Table 6; Fig. 4c). Although not statistically significant, the pooled remission rates tended to be higher after rTMS relative to DTMS in studies with the open-label designs only and in studies with patients on concurrent antidepressants (Table 6; Additional file 1: Figure S11).

## Discussion

The current focus of research in the field of the non-invasive brain stimulation is concerned with identifying predictors of response [34] to determine which variants of these methods (conventional F8-coils, H1-coils, or other systems) are most effective in the treatment of MDD [10]. Although F8-coils and H1-coils are FDA-approved for treatment-resistant MDD, surprisingly little is still known about the most optimal stimulation protocols required for best acute and longer-term efficacy of these methods. Although not designed to assess efficacy, the current study provides the first approach to systematically assess and compare the antidepressant outcomes of rTMS with F8-coil and DTMS with H1-coil in unipolar MDD in studies matched on stimulation frequency.

The overall assessment of antidepressant outcomes with either coil (F8-coil and H1-coil) highlights the following main findings. First, the primary outcome (depression severity) was alleviated following the high frequency (18–20 Hz) stimulation with either coil (F8-coil and H1-coil) already after 10 daily sessions relative to baseline in unipolar MDD. However, other meta-analyses have shown that depression severity was reduced even further at session 20 relative to session 10 in rTMS studies with F8-coils [35] or in DTMS studies with H1-coils [36]. Similar, response and remission rates are relatively low after 10 sessions of rTMS or DTMS (29 and 15%, respectively) in the current analysis. Both rates were shown to increase after 20 relative to 10 sessions of DTMS [36]. Therefore, at least 20 daily stimulation sessions may be required for clinically (rather than statistically) meaningful effects of rTMS or DTMS in the acute treatment of unipolar MDD. The open-label extensions to the largest RCTs in the field [2, 9] have shown that some patients experience a delayed response and require 4–6 weeks of stimulation before showing adequate response to treatment with F8-coil or H1-coil [37, 38]. The downside of prolonging treatment is the issue of higher costs and inconvenience of treatment leading to higher dropout rates. One alternative approach in the field aiming to reduce these problems is to provide an accelerated treatment with more than one daily session to induce efficacy faster [39]. However, such protocols still need to be tested in larger RCTs and compared to the single-sessions per day using head-to-head designs.

Second, the primary outcome (depression severity) did not depend on the study design (RCT vs. open-label) nor the therapy type (add-on to antidepressants vs. monotherapy). However, our results suggest that the reduction in depression severity was lower in studies with older patients, as also reported by others [40]. This result could be due to reduced plasticity, connectivity, and the motor threshold in older patients. The effect of age could also depend on the length or the severity of illness that may be higher in older patients. Therefore, future research needs to devise protocols tailored towards the needs of older patients and/or patients with higher illness severity. For instance, older patient groups may benefit most from high intensity stimulation with a large number of pulses due to lower capacity for plasticity as the brain ages [41].

Third, the secondary outcomes (response and remission rates) after the stimulation with either coil (F8-coil and H1-coil) were low. Since these outcomes are not controlled for inactive sham, it is unclear if they are comparable to response and remission rates in RCTs conducted with either coil. The secondary outcomes tended to increase in the open-label studies relative to the blinded RCTs and in studies with patients receiving concurrent antidepressants relative to the monotherapy. These results probably reflect real-world outcomes whereby patients in the clinical practice know what treatment they receive and for ethical reasons are kept on medication even if they do not adequately respond to such pharmacotherapy [8, 42]. Although the current response and remission rates following the active stimulation with either coil are not controlled for sham, these effects cannot be explained by placebo/expectation alone because active stimulation produced better efficacy than inactive sham in several RCTs with the F8-coil [1] and the H1-coil [9]. In general, the efficacy of both methods (F8-coil and H1-coil) with highly variable stimulation protocols is comparable in magnitude based on the moderate acute effects observed in the double-blind, sham-controlled RCTs with MDD patients [1, 3, 9, 43]. In the same vein, the current response and remission rates cannot be attributed to antidepressants alone given that patients in most studies were pharmacoresistant and efficacy was demonstrated in RCTs with F8-coil and H1-coil applied as the monotherapy for MDD [3, 9]. It has been suggested that brain stimulation may enhance or precede subsequent effects of antidepressants for some patients, for instance by rendering neural pathways more susceptible to drug-induced functional plasticity [43].

The comparison of rTMS studies with F8-coil and DTMS studies with H1-coil suggests that there might be some differences in the antidepressant outcomes of these two methods. Our results indicate that the open-label H1-coil studies demonstrated a consistently larger reduction in the primary outcome (depression severity) relative to the F8-coil in all studies. This pattern was also observed when only comparing H-coil and F8-coil studies in open-label designs and in studies with patients concurrently receiving antidepressants, and was thus irrespective of the study design (RCT and open-label) and the therapy type (add-on to antidepressants or monotherapy). In contrast, any differences in the secondary outcomes (response and remission rates) were less consistent. The remission rates tended to favour the F8-coil relative to the H1-coil in all studies, while all other comparisons, including remission and response rates were not statistically significant. The visual inspection of forest plots (Fig. 4) also did not reveal any consistent trends towards differences in the secondary outcomes between both coils. It is unclear if the differences in the primary outcome between the F8-coil and the H1-coil are meaningful (clinically-relevant) or if they are secondary to various factors not controlled for in our study. For example, the H1-coil may reduce depression severity faster than the F8-coil (already after 10 daily stimulation sessions) while the comparison of the same outcome at a later session (20 or later) could reveal no differences between both coils. Such a comparison was not conducted here since most of the rTMS studies did not continue for longer than 10 sessions with the extra-high frequency of 18–20 Hz. The current results suggest that even a large reduction in depression scores may be inadequate to classify the patients as responders or remitters in case their baseline depression severity is high and/or if response and remission require stimulation for more than 10 daily sessions. Thus, not the coils themselves but rather factors, such as time of assessment, baseline depression severity as well as definitions of response and remission based on different versions of HDRS might have contributed to the inconsistent secondary outcome scores observed in this analysis.

It is not surprising that the non-focal stimulation with the H1-coil produces larger reduction in depression severity than the more focal F8-coil, particularly since the target region is difficult to locate in the rTMS studies with the F8-coil [44]. The fact that the more widespread stimulation is likely to target the appropriate region may in itself be an advantage of the H1-coil. In addition, DTMS with H1-coil may also directly influence the activity of deeper, subcortical regions in the emotion regulation network [45]. However, to date it is still being debated whether DTMS is indeed as deep as originally proposed [46] or whether it induces its effects mainly by stimulating a less focal cortical surface relative to a focal stimulation delivered with the F8-coil [47]. A recent mathematical model suggests that relative to 2.2 cm with the F8-coil, the H1-coil can reach up to 6.4 cm while retaining 50% of its maximum electrical field strength [45]. Future investigations by independent groups should corroborate these findings. Finally, the stimulation intensity as well as other parameters not controlled for in this study might have contributed to the greater reduction in depression severity in studies with H1-coil relative to studies with F8-coil. In general, the DTMS studies with H1-coil utilised the high intensity protocols (120% MT, 1680–1980 stimuli/session) relative to the lower intensity protocols (80–110% MT, 800–2000 stimuli/session) in the rTMS studies with F8-coil. However, intensity alone was not related to depression severity in the rTMS studies meaning that a combination of parameters may be required for the most optimal antidepressant outcomes. Since two meta-analyses showed that lower number of stimuli/session (1200–1500) produced greater reduction in depression severity in rTMS studies with F8-coil [3, 35], not intensity alone but rather the combination of frequency-intensity-stimuli may be predictive of antidepressant outcomes. One caveat of the high frequency/intensity of stimulation is the trend towards higher dropout rates in DTMS relative to rTMS studies and the increased risk of seizures with H1-coils due to the larger volume of stimulated tissue [48]. Indeed, two seizures were reported in the DTMS studies with H1-coil and none in the rTMS studies with F8-coil. More research is also required to investigate if the length of the inter-train-interval could also be a predictive factor for the antidepressant outcomes of the F8-coil and the H1-coil.

The systematic assessment of the literature shows that the rTMS studies with F8-coil apply highly variable stimulation protocols to highly heterogeneous groups of patients since 1997 [3]. Although the stimulation protocols are more consistent in DTMS studies with the H1-coil, our analysis did not identify predictors of antidepressant outcomes apart from patient age that may be confounded by duration and severity of illness. This may be a result of a low statistical power in a meta-analysis with data from only 19 studies. However, it is also in line with previous studies that did not consistently identify predictors of response to rTMS or DTMS using primary and secondary analyses [3, 37, 49, 50]. The use of multivariate regression models that probe the effect of one predictor on multiple outcomes while holding all other factors constant is required once an adequate volume of data exist.

It remains unclear for how long the antidepressant outcomes of the non-invasive brain stimulation last in MDD [51]. Given a moderate efficacy to any kind of non-invasive brain stimulation [52], both rTMS and DTMS are viable alternatives for patients resistant to pharmacotherapy in the short-term. Findings regarding the durability of the antidepressant outcomes of both DTMS and rTMS indicate that such effects may last up to six months to a year in some cases [51, 53]. In particular, regular maintenance treatments may prolong the acute antidepressant outcomes and prevent relapse [4, 20]. However, these findings also highlight substantial variability among patients. Maintenance may have to be based on individual patients’ needs as to optimise cost-effectiveness for prevention of relapse.

According to discussions at the 2nd European Conference on Brain Stimulation in Psychiatry (ECBSP, October 2017, Munich, Germany), the important focus in the field of the non-invasive brain stimulation is the individualisation of treatment. However, given the vast options regarding the parameters of stimulation, identifying an optimal protocol may be akin to finding the proverbial needle in the haystack. One way of constraining the number of possibilities is by understanding the factors that influence neuronal communication and in turn plasticity [54, 55]. In fact, such an approach has been used to guide the design of various TMS methods in the past, such as theta burst stimulation [56]. It is also currently being explored to tailor TMS methods in a way that may either interfere with the abnormal or boost the beneficial cortical oscillations in a frequency-specific manner [57, 58]. Such applications may be tailored to an individual’s endogenous neuronal activity but do not have a substantial evidence base for the therapeutic use in MDD yet. Answering these outstanding questions regarding the optimal design of a treatment course is challenging because the demographic and clinical characteristics of patients might also influence the outcomes of studies irrespective of the stimulation protocols, particularly when sample sizes are small.

The present meta-analysis has several limitations. First, the studies selected here may not be representative of the large body of literature available in this field with respect to the stimulation protocols and the primary diagnoses. In the attempt to homogenise the study parameters, we focus on one stimulation frequency (10 Hz) and one outcome assessment point (after 10 sessions) rather than the clinically-approved stimulation protocols. Furthermore, studies with mixed unipolar and bipolar MDD samples were excluded since the antidepressant outcomes may differ in patients with unipolar vs. bipolar MDD. Second, the current meta-analysis is based on the outcomes of active stimulation in all studies because some studies did not have sham control groups. Although the study design (RCT vs. open-label) had little effect on the primary outcomes (depression severity), the secondary outcomes (response and remission rates) tended to be inflated in the open-label studies with either coil. Such inflated effects might have resulted from the potentially high risk of selection, performance, and detection bias in the open-label studies because no other risks were identified in all studies using the Cochrane tool [59] (Additional file 1: Table S1). Therefore, the antidepressant outcomes reported here cannot be used to quantify the magnitude of the clinical efficacy of either coil. Third, there was too little information regarding safety of both methods reported at session 10 of treatment. Thus, we could not analyse the drop-out rates meta-analytically in the current analysis. Fourth, it was not possible to match the rTMS and the DTMS studies based on the number of pulses as well as other stimulation parameters, such as intensity and inter-train-interval. It is likely that the antidepressant outcomes of either coil depend on a complex interaction between various stimulation parameters and patient characteristics [11]. Fifth, the antidepressant outcomes are based on various versions of HDRS and consequently on different definitions (cut-off scores) of the secondary outcomes (response and remission rates). Relative to the primary outcome, the analyses regarding the secondary outcomes are also based on a lower volume of data with unequal number of open-label studies and RCTs for each coil. These factors might have masked any real differences in the secondary outcomes between both coils. Sixth, our comparison of antidepressant outcomes is based on the between-study rather than the within-study effects. Research conducted independently of the companies producing various non-invasive brain stimulation devices is required to compare the efficacy of such coils in head-to-head designs. Finally, we have not assessed the antidepressant outcomes of FDA-approved clinical protocols of rTMS or DTMS and instead focused on the same frequency of stimulation (18–20 Hz). The focus on stimulation frequency contributed to exclusion of majority of rTMS studies since rTMS with F8-coil is most often conducted using the frequency of 10 Hz rather than 20 Hz [3] while 18–20 Hz is used in all DTMS protocols in MDD studies to date. The overwhelming volume of evidence suggests that rTMS or DTMS with higher frequencies produces greater beneficial effects by alleviating symptom severity and improving cognition in MDD [34, 60, 61]. However, high frequencies are also associated with the risk of seizures, scalp discomfort, and headaches [62]. In general, a definition of ‘high-frequency’ stimulation is highly variable and includes any frequency above 1 Hz [34]. As a consequence, the high-frequency protocols in rTMS studies have also utilised a wide range of frequencies (e.g. 10, 15, and 20 Hz), while DTMS has been more restrictive in terms of frequency range (18–20 Hz). Although 10 Hz stimulation is being most often used in the clinic when applying rTMS with the F8-coil, it is unclear which of these high frequencies is most effective when controlling for other potentially confounding variables. Our results suggest that each coil may require an individual combination of parameters for the most optimal antidepressant outcomes for individual patients.

## Conclusion

When matched on the frequency of stimulation, rTMS with F8-coil and DTMS with H1-coil produced large, acute reduction in depression severity at session 10 relative to baseline in unipolar MDD. The reduction in depression severity was greater in studies with younger patients. The comparison between coils showed a larger reduction in depression severity in H1-coil vs. F8-coil studies (independent of the study design or the concurrent pharmacotherapy) and a trend towards higher remission rates in F8-coil vs. H1-coils studies. These effects are based on a low volume of studies, are not controlled for placebo, and may not be clinically-relevant. The stimulation protocols differed systematically because stimulation was more focal but less intense (80–110% of the resting motor threshold, MT) in the F8-coil studies and less focal but more intense (120% MT) in the H1-coil studies. Head-to-head trials are required to compare the antidepressant outcomes of rTMS with F8-coil and DTMS with H1-coil to identify the most optimal stimulation protocols for acute and longer-lasting efficacy.

## Additional files

Additional file 1: **Figure S1.** Depression severity: outlier analysis and one-study removed analysis. **Figure S2.** Depression severity: subgroup analysis. **Figure S3.** Depression severity: publication bias analysis. **Figure S4.** Response rates: subgroup analysis. **Figure S5.** Response rates: publication bias analysis. **Figure S6.** Remission rates: subgroup analysis. **Figure S7.** Remission rates: meta-regression analysis. **Figure S8.** Remission rates: publication bias analysis. **Figure S9.** Depression severity: subgroup analysis (H1-coil vs. F8-coil). **Figure S10.** Response rates: subgroup analysis (H1-coil vs. F8-coil). **Figure S11.** Remission rates: subgroup analysis (H1-coil vs. F8-coil). **Table S1.** Risk of bias assessment. (PDF 165 kb)

## Abbreviations

*b*: Unstandardised weighted regression coefficient
*CI*: 95% confidence interval
*df*: Degrees of freedom
DLPFC: Dorsolateral prefrontal cortex
DTMS: Deep transcranial magnetic stimulation
ECT: Electroconvulsive therapy
F8: Figure-of-eight coil (rTMS)
*g*: Hedges’ *g* (standardised paired difference in means corrected for the sample size)
H1: H1-coil (DTMS)
HDRS: Hamilton Depression Rating Scale
HF-rTMS: High-frequency repetitive transcranial magnetic stimulation
*k*: Number of studies
L: Left PFC
LOCF: Last observation carried forward
MDD: Major depressive disorder
MT: Motor threshold
*n*: Sample size
PFC: Prefrontal cortex
RCT: Randomised-controlled trial
rTMS: Repetitive transcranial magnetic stimulation
*SD*: Standard deviation
TI: Title
TMS: Transcranial magnetic stimulation
TX: Text
