# Supplementary material for: Antidepressant outcomes of high-frequency repetitive transcranial magnetic stimulation (rTMS) with F8-coil and deep transcranial magnetic stimulation (DTMS) with H1-coil in major depression: a systematic review and meta-analysis
Source: BMC Psychiatry. 2019 May 7;19:139. doi: 10.1186/s12888-019-2106-7 (PMC6505129; doi:10.1186/s12888-019-2106-7)
Supplement: Supplementary file 1 — Figure S1. Depression severity: outlier analysis and one-study removed analysis. Figure S2. Depression severity: subgroup analysis. Figure S3. Depression severity: publication bias analysis. Figure S4. Response rates: subgroup analysis. Figure S5. Response rates: publication bias analysis. Figure S6. Remission rates: subgroup analysis. Figure S7. Remission rates: meta-regression analysis. Figure S8. Remission rates: publication bias analysis. Figure S9. Depression severity: subgroup analysis (H1-coil vs. F8-coil). Figure S10. Response rates: subgroup analysis (H1-coil vs. F8-coil). Figure S11. Remission rates: subgroup analysis (H1-coil vs. F8-coil). Table S1. Risk of bias assessment. (PDF 165 kb) [file 12888_2019_2106_MOESM1_ESM.pdf]

Gellersen, HM, Kedzior, KK. Antidepressant outcomes of high-frequency repetitive transcranial magnetic stimulation (rTMS) with F8-coil and deep transcranial magnetic stimulation (DTMS) with H1-coil in major depression: a systematic review and meta-analysis. BMC Psychiatry. 2019; doi: https://doi.org/10.1186/s12888-019-2106-7

Table of Contents

Studies in meta-analysis 2
A. DTMS with H1-coil (*k*=8) 2
B. rTMS with F8-coil (*k*=12) 2
Figure S1. Depression severity: outlier analysis and one-study removed analysis 3
A. Outlier analysis 3
B. One-study removed analysis 3
Figure S2. Depression severity: subgroup analysis 5
A. Study design (RCT vs. open-label) 5
B. Therapy type (add-on to antidepressants vs. monotherapy) 6
Figure S3. Depression severity: publication bias analysis 7
Figure S4. Response rates: subgroup analysis 8
A. Study design (RCT vs. open-label) 8
B. Therapy type (add-on to antidepressants vs. monotherapy) 9
Figure S5. Response rates: publication bias analysis 10
Figure S6. Remission rates: subgroup analysis 11
A. Study design (RCT vs. open-label) 11
B. Therapy type (add-on to antidepressants vs. monotherapy) 12
Figure S7. Remission rates: meta-regression analysis 13
Figure S8. Remission rates: publication bias analysis 14
Figure S9. Depression severity: subgroup analysis (H1-coil vs. F8-coil) 15
A. Open-label studies 15
B. Add-on studies 16
Figure S10. Response rates: subgroup analysis (H1-coil vs. F8-coil) 17
A. Open-label studies 17
B. Add-on studies 18
Figure S11. Remission rates: subgroup analysis (H1-coil vs. F8-coil) 19
A. Open-label studies 19
B. Add-on studies 20
Table S1. Risk of bias assessment 21
References 22

Studies in meta-analysis

k=19 with *k*=20 independent subgroups

A. DTMS with H1-coil (*k*=8)

Levkovitz et al., 2009 [1]
Rosenberg et al., 2010a [2]
Rosenberg et al., 2010b [3]
Isserles et al., 2011 [4]
Harel et al., 2014 [5]
Levkovitz et al., 2015 [6]
Rapinesi et al., 2015a [7]
Rapinesi et al., 2015b [8]

B. rTMS with F8-coil (*k*=12)

George et al., 1997 [9]
Berman et al., 2000 [10]
Catafau et al., 2001 [11]
Garcia-Toro et al., 2001a [12]
Garcia-Toro et al., 2001b [13]
Boutros et al., 2002 [14]
Bajbouj et al., 2005 [15]
Yukimasa et al., 2006 [16]
Luborzewski et al., 2007 [17]
Bakim et al., 2012a [18] 80%
Bakim et al., 2012b [18] 110%
Chen et al., 2013 [19]

Figure S1. Depression severity: outlier analysis and one-study removed analysis

A. Outlier analysis

Group by Outcome	Study name	Outcome	Hedges's g	Lower limit	Upper limit	p-Value	Total	Std Residual	P-Val
F8	George et al., 1997	F8	0.78	0.02	1.54	0.045	7	-0.39	0.695
F8	Berman et al., 2000	F8	1.15	0.39	1.91	0.003	10	0.36	0.718
F8	Catafau et al., 2001	F8	0.31	-0.42	1.03	0.407	5	-1.39	0.165
F8	Garcia-Toro et al., 2001a	F8	1.02	0.45	1.58	0.000	17	0.10	0.917
F8	Garcia-Toro et al., 2001b	F8	1.28	0.52	2.04	0.001	11	0.62	0.534
F8	Boutros et al., 2002	F8	0.89	0.26	1.53	0.006	12	-0.18	0.859
F8	Bajbouj et al., 2005	F8	0.95	0.53	1.38	0.000	30	-0.06	0.955
F8	Yukimasa et al., 2006	F8	0.78	0.35	1.20	0.000	26	-0.52	0.600
F8	Luborzewski et al., 2007	F8	0.59	0.10	1.09	0.019	17	-0.96	0.336
F8	Bakim et al., 2012, 80%	F8	1.23	0.51	1.95	0.001	12	0.54	0.589
F8	Bakim et al., 2012, 110%	F8	1.48	0.66	2.31	0.000	11	0.98	0.326
F8	Chen et al., 2013	F8	6.40	3.54	9.26	0.000	10	3.64	0.000
F8			0.97	0.70	1.25	0.000			
H1	Levkovitz et al., 2009	H1	1.65	0.98	2.31	0.000	20	0.20	0.844
H1	Rosenberg et al., 2010a	H1	1.48	0.47	2.49	0.004	7	-0.12	0.907
H1	Rosenberg et al., 2010b	H1	1.94	0.65	3.23	0.003	6	0.52	0.601
H1	Isserles et al., 2011	H1	2.36	1.52	3.20	0.000	20	1.47	0.142
H1	Harel et al., 2014	H1	1.61	1.04	2.19	0.000	26	0.14	0.890
H1	Levkovitz et al., 2015	H1	1.12	0.85	1.40	0.000	83	-1.16	0.247
H1	Rapinesi et al., 2015a	H1	2.71	1.33	4.09	0.000	9	1.48	0.140
H1	Rapinesi et al., 2015b	H1	0.84	0.21	1.46	0.009	12	-1.53	0.125
H1			1.55	1.17	1.94	0.000			

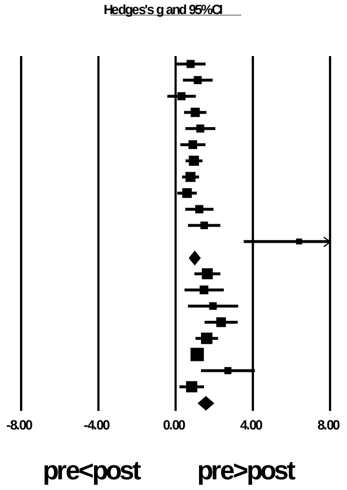

B. One-study removed analysis

Study name	Outcome	Point	Lower limit	Upper limit	p-Value
Levkovitz et al., 2009	H1	1.17	0.93	1.42	0.000
Rosenberg et al., 2010a	H1	1.19	0.95	1.44	0.000
Rosenberg et al., 2010b	H1	1.18	0.94	1.42	0.000
Isserles et al., 2011	H1	1.14	0.91	1.36	0.000
Harel et al., 2014	H1	1.17	0.93	1.42	0.000
Levkovitz et al., 2015	H1	1.22	0.96	1.49	0.000
Rapinesi et al., 2015a	H1	1.16	0.93	1.39	0.000
Rapinesi et al., 2015b	H1	1.23	0.98	1.47	0.000
George et al., 1997	F8	1.22	0.98	1.47	0.000
Berman et al., 2000	F8	1.21	0.96	1.45	0.000
Catafau et al., 2001	F8	1.24	1.01	1.48	0.000
Garcia-Toro et al., 2001a	F8	1.22	0.97	1.47	0.000
Garcia-Toro et al., 2001b	F8	1.20	0.95	1.45	0.000
Boutros et al., 2002	F8	1.22	0.97	1.47	0.000
Bajbouj et al., 2005	F8	1.23	0.97	1.48	0.000
Yukimasa et al., 2006	F8	1.24	0.99	1.49	0.000
Luborzewski et al., 2007	F8	1.24	1.00	1.48	0.000
Bakim et al., 2012, 80%	F8	1.20	0.95	1.45	0.000
Bakim et al., 2012, 110%	F8	1.19	0.94	1.43	0.000
Chen et al., 2013	F8	1.15	0.94	1.35	0.000
		1.20	0.96	1.44	0.000

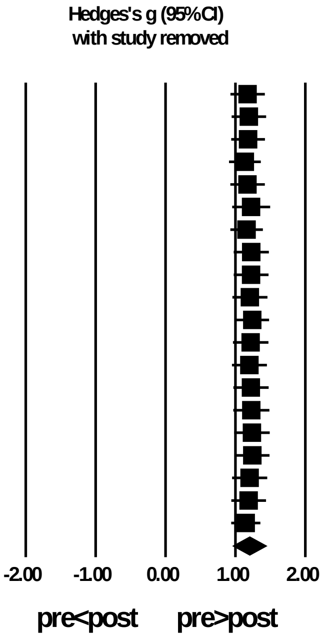

Note. Figures A-B are forest plots of the random-effects meta-analyses of the primary antidepressant outcome (depression severity at baseline (pre) – session 10 (post)) in all studies with either coil (F8-coil and H1-coil). Figure S1A shows the results of an outlier analysis. The effect size (Hedges' *g*) in one study (Chen et al., 2013) was significantly ($p<.01$) higher than all other effects in rTMS studies with F8-coil. The removal of this study did not change the interpretation of the pooled mean weighted effect that was 1.20 with and 1.15 without the study by Chen et al., 2013, respectively (Figure S1B).

Abbreviations: *CI*, 95% confidence interval; DTMS, deep transcranial magnetic stimulation; F8, figure-of-eight coil (rTMS); H1, H1-coil (DTMS); Hedges' *g* (effect size), standardised paired difference in means corrected for the sample size; rTMS, repetitive transcranial magnetic stimulation; Total, sample size per study.

Figure S2. Depression severity: subgroup analysis

A. Study design (RCT vs. open-label)

Group by Study design	Study name	Outcome	Statistics for each study: Hedges's g	Lower limit	Upper limit	p-Value	Total
open-label	Levkovitz et al., 2009	H1	1.65	0.98	2.31	0.000	20
open-label	Rosenberg et al., 2010a	H1	1.48	0.47	2.49	0.004	7
open-label	Rosenberg et al., 2010b	H1	1.94	0.65	3.23	0.003	6
open-label	Isserles et al., 2011	H1	2.36	1.52	3.20	0.000	20
open-label	Harel et al., 2014	H1	1.61	1.04	2.19	0.000	26
open-label	Rapinesi et al., 2015a	H1	2.71	1.33	4.09	0.000	9
open-label	Rapinesi et al., 2015b	H1	0.84	0.21	1.46	0.009	12
open-label	Catafau et al., 2001	F8	0.31	-0.42	1.03	0.407	5
open-label	Bajbouj et al., 2005	F8	0.95	0.53	1.38	0.000	30
open-label	Yukimasa et al., 2006	F8	0.78	0.35	1.20	0.000	26
open-label	Luborzewski et al., 2007	F8	0.59	0.10	1.09	0.019	17
open-label			1.24	0.87	1.61	0.000	
RCT	Levkovitz et al., 2015	H1	1.12	0.85	1.40	0.000	83
RCT	George et al., 1997	F8	0.78	0.02	1.54	0.045	7
RCT	Berman et al., 2000	F8	1.15	0.39	1.91	0.003	10
RCT	Garcia-Toro et al., 2001a	F8	1.02	0.45	1.58	0.000	17
RCT	Garcia-Toro et al., 2001b	F8	1.28	0.52	2.04	0.001	11
RCT	Boutros et al., 2002	F8	0.89	0.26	1.53	0.006	12
RCT	Bakim et al., 2012, 80%	F8	1.23	0.51	1.95	0.001	12
RCT	Bakim et al., 2012, 110%	F8	1.48	0.66	2.31	0.000	11
RCT	Chen et al., 2013	F8	6.40	3.54	9.26	0.000	10
RCT			1.17	0.86	1.48	0.000	

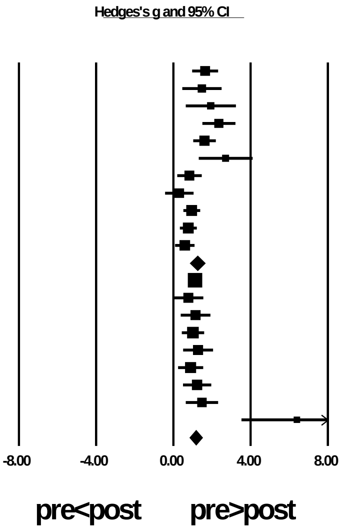

B. Therapy type (add-on to antidepressants vs. monotherapy)

Group by Any antidepressants	Study name	Outcome	Hedges's g	Lower limit	Upper limit	p-Value	Total
no	Levkovitz et al., 2009	H1	1.65	0.98	2.31	0.000	20
no	Rosenberg et al., 2010a	H1	1.48	0.47	2.49	0.004	7
no	Levkovitz et al., 2015	H1	1.12	0.85	1.40	0.000	83
no	Berman et al., 2000	F8	1.15	0.39	1.91	0.003	10
no			1.21	0.98	1.44	0.000	
yes	Rosenberg et al., 2010b	H1	1.94	0.65	3.23	0.003	6
yes	Isserles et al., 2011	H1	2.36	1.52	3.20	0.000	20
yes	Harel et al., 2014	H1	1.61	1.04	2.19	0.000	26
yes	Rapinesi et al., 2015a	H1	2.71	1.33	4.09	0.000	9
yes	Rapinesi et al., 2015b	H1	0.84	0.21	1.46	0.009	12
yes	George et al., 1997	F8	0.78	0.02	1.54	0.045	7
yes	Catafau et al., 2001	F8	0.31	-0.42	1.03	0.407	5
yes	Garcia-Toro et al., 2001a	F8	1.02	0.45	1.58	0.000	17
yes	Garcia-Toro et al., 2001b	F8	1.28	0.52	2.04	0.001	11
yes	Boutros et al., 2002	F8	0.89	0.26	1.53	0.006	12
yes	Bajbouj et al., 2005	F8	0.95	0.53	1.38	0.000	30
yes	Yukimasa et al., 2006	F8	0.78	0.35	1.20	0.000	26
yes	Luborzewski et al., 2007	F8	0.59	0.10	1.09	0.019	17
yes	Bakim et al., 2012, 80%	F8	1.23	0.51	1.95	0.001	12
yes	Bakim et al., 2012, 110%	F8	1.48	0.66	2.31	0.000	11
yes	Chen et al., 2013	F8	6.40	3.54	9.26	0.000	10
yes			1.20	0.89	1.50	0.000	
Overall			1.21	1.02	1.39	0.000	

Hedges's g and 95%CI

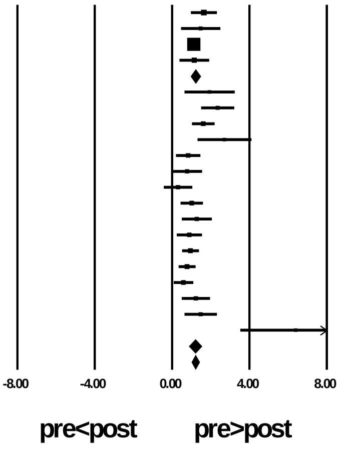

Note. Figures A-B are forest plots of mixed-effects meta-analyses comparing the antidepressant outcomes (depression severity) based on the study designs (RCTs vs. open-label, Figure A) or the therapy type (add-on to antidepressants vs. monotherapy, Figure B) in studies with either coil (F8-coil and H1-coil).

Abbreviations: *CI*, 95% confidence interval; DTMS, deep transcranial magnetic stimulation; F8, figure-of-eight coil (rTMS); H1, H1-coil (DTMS); Hedges' *g* (effect size), standardised paired difference in means corrected for the sample size; rTMS, repetitive transcranial magnetic stimulation; Total, sample size per study.

Figure S3. Depression severity: publication bias analysis

Classic fail-safe N

Z-value for observed studies	16.34911
P-value for observed studies	0.00000
Alpha	0.05000
Tails	2.00000
Z for alpha	1.95996
Number of observed studies	20.00000
Number of missing studies that would bring p-value to > alpha	1372.00000

Orwin's fail-safe N

Hedges's g in observed studies	1.10522
Criterion for a 'trivial' hedges's g	0.20000
Mean hedges's g in missing studies	0.10000
Number missing studies needed to bring hedges's g under 0.2	182.00000

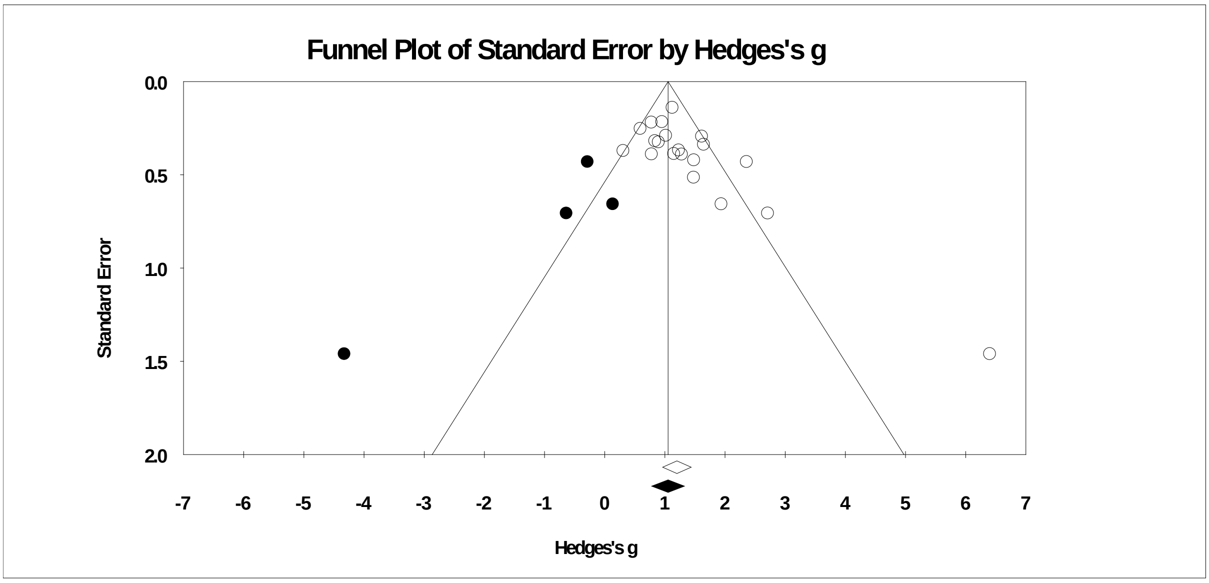

Note. The funnel plot shows a distribution of all effect sizes (the unfilled circles) around the pooled mean weighted effect of all studies (the unfilled diamond). Four studies theoretically missing from the analysis (the filled circles) are required to make the plot symmetric. The interpretation of the analysis including the missing studies (the filled diamond) is the same as without these studies (the unfilled diamond). If the missing studies indicate that publication bias occurred, such bias has no effect on the interpretation of the results of this analysis.

Figure S4. Response rates: subgroup analysis

A. Study design (RCT vs. open-label)

Group by Study design	Study name	Outcome	Event rate	Lower limit	Upper limit	Total
open-label	Levkovitz et al., 2009	H1	0.45	0.25	0.66	9 / 20
open-label	Rosenberg et al., 2010a	H1	0.29	0.07	0.67	2 / 7
open-label	Rosenberg et al., 2010b	H1	0.50	0.17	0.83	3 / 6
open-label	Rapinesi et al., 2015a	H1	0.05	0.00	0.47	0 / 9
open-label	Rapinesi et al., 2015b	H1	0.04	0.00	0.40	0 / 12
open-label	Catafau et al., 2001	F8	0.40	0.10	0.80	2 / 5
open-label	Bajbouj et al., 2005	F8	0.33	0.19	0.52	10 / 30
open-label	Yukimasa et al., 2006	F8	0.19	0.08	0.39	5 / 26
open-label	Luborzewski et al., 2007	F8	0.35	0.17	0.60	6 / 17
open-label			0.32	0.23	0.42	
RCT	Levkovitz et al., 2015	H1	0.15	0.09	0.24	13 / 89
RCT	George et al., 1997	F8	0.14	0.02	0.58	1 / 7
RCT	Berman et al., 2000	F8	0.10	0.01	0.47	1 / 10
RCT	Garcia-Toro et al., 2001b	F8	0.36	0.14	0.66	4 / 11
RCT	Boutros et al., 2002	F8	0.25	0.08	0.55	3 / 12
RCT	Chen et al., 2013	F8	0.70	0.38	0.90	7 / 10
RCT			0.26	0.13	0.47	

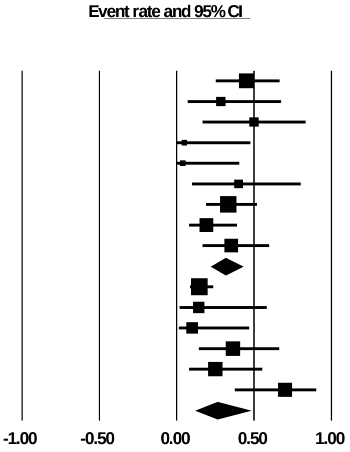

B. Therapy type (add-on to antidepressants vs. monotherapy)

Group by Any antidepressants	Study name	Outcome	Event rate	Lower limit	Upper limit	Total
no	Levkovitz et al., 2009	H1	0.45	0.25	0.66	9 / 20
no	Rosenberg et al., 2010a	H1	0.29	0.07	0.67	2 / 7
no	Levkovitz et al., 2015	H1	0.15	0.09	0.24	13 / 89
no	Berman et al., 2000	F8	0.10	0.01	0.47	1 / 10
no			0.23	0.10	0.45	
yes	Rosenberg et al., 2010b	H1	0.50	0.17	0.83	3 / 6
yes	Rapinesi et al., 2015a	H1	0.05	0.00	0.47	0 / 9
yes	Rapinesi et al., 2015b	H1	0.04	0.00	0.40	0 / 12
yes	George et al., 1997	F8	0.14	0.02	0.58	1 / 7
yes	Catafau et al., 2001	F8	0.40	0.10	0.80	2 / 5
yes	Garcia-Toro et al., 2006b	F8	0.36	0.14	0.66	4 / 11
yes	Boutros et al., 2002	F8	0.25	0.08	0.55	3 / 12
yes	Bajbouj et al., 2005	F8	0.33	0.19	0.52	10 / 30
yes	Yukimasa et al., 2006	F8	0.19	0.08	0.39	5 / 26
yes	Luborzewski et al., 2007	F8	0.35	0.17	0.60	6 / 17
yes	Chen et al., 2013	F8	0.70	0.38	0.90	7 / 10
yes			0.31	0.22	0.43	

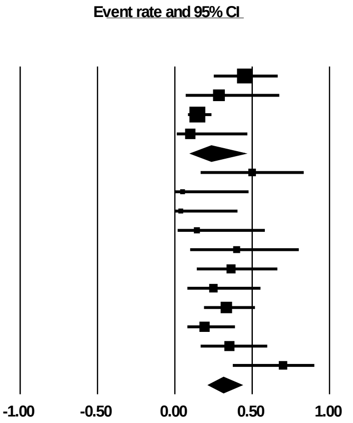

Note. Figures A-B are forest plots of mixed-effects meta-analyses comparing the antidepressant outcomes (response rates) based on the study designs (RCTs vs. open-label, Figure A) or the therapy type (add-on to antidepressants vs. monotherapy, Figure B) in studies with either coil (F8-coil and H1-coil).

Abbreviations: *CI*, 95% confidence interval; DTMS, deep transcranial magnetic stimulation; F8, figure-of-eight coil (rTMS); H1, H1-coil (DTMS); rTMS, repetitive transcranial magnetic stimulation; Total, sample size per study.

Figure S5. Response rates: publication bias analysis

Classic fail-safe N

Z-value for observed studies	-5.95264
P-value for observed studies	0.00000
Alpha	0.05000
Tails	2.00000
Z for alpha	1.95996
Number of observed studies	15.00000
Number of missing studies that would bring p-value to > alpha	124.00000

Orwin's fail-safe N

Event rate in observed studies	0.27178
Criterion for a 'trivial' event rate	0.10000
Mean event rate in missing studies	0.05000
Number missing studies needed to bring event rate under 0.1	25.00000

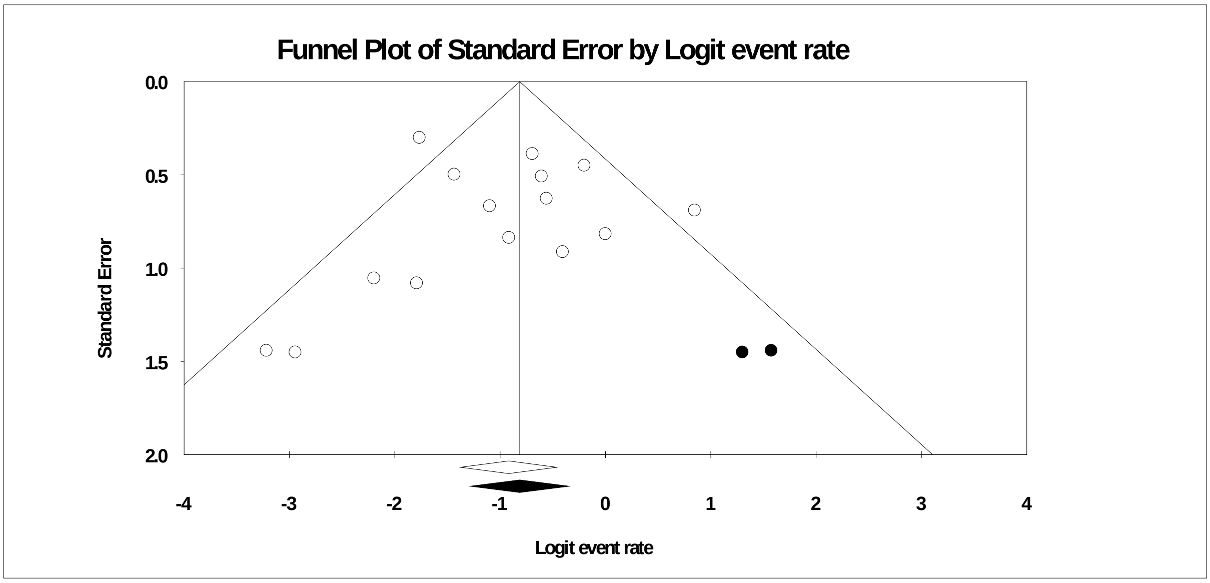

Note. The funnel plot shows a distribution of all effect sizes (the unfilled circles) around the pooled mean weighted effect of all studies (the unfilled diamond). Two studies theoretically missing from the analysis (the filled circles) are required to make the plot symmetric. The interpretation of the analysis including the missing studies (the filled diamond) is the same as without these studies (the unfilled diamond). If the missing studies indicate that publication bias occurred, such bias has no effect on the interpretation of the results of this analysis.

Figure S6. Remission rates: subgroup analysis

A. Study design (RCT vs. open-label)

Group by Study design	Study name	Outcome	Event rate	Lower limit	Upper limit	Total
open-label	Levkovitz et al., 2009	H1	0.20	0.08	0.43	4 / 20
open-label	Rosenberg et al., 2010a	H1	0.14	0.02	0.58	1 / 7
open-label	Rosenberg et al., 2010b	H1	0.17	0.02	0.63	1 / 6
open-label	Rapinesi et al., 2015a	H1	0.11	0.02	0.50	1 / 9
open-label	Rapinesi et al., 2015b	H1	0.04	0.00	0.40	0 / 12
open-label	Catafau et al., 2001	F8	0.08	0.01	0.62	0 / 5
open-label	Yukimasa et al., 2006	F8	0.27	0.13	0.47	7 / 26
open-label	Luborzewski et al., 2007	F8	0.29	0.13	0.54	5 / 17
open-label			0.21	0.14	0.31	
RCT	Levkovitz et al., 2015	H1	0.07	0.03	0.14	6 / 89
RCT	George et al., 1997	F8	0.14	0.02	0.58	1 / 7
RCT	Berman et al., 2000	F8	0.10	0.01	0.47	1 / 10
RCT	Boutros et al., 2002	F8	0.08	0.01	0.41	1 / 12
RCT			0.08	0.04	0.14	

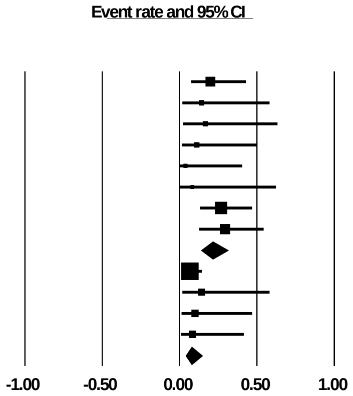

B. Therapy type (add-on to antidepressants vs. monotherapy)

Group by Any antidepressants	Study name	Outcome	Event rate	Lower limit	Upper limit	Total
no	Levkovitz et al., 2009	H1	0.20	0.08	0.43	4 / 20
no	Rosenberg et al., 2010a	H1	0.14	0.02	0.58	1 / 7
no	Levkovitz et al., 2015	H1	0.07	0.03	0.14	6 / 89
no	Berman et al., 2000	F8	0.10	0.01	0.47	1 / 10
no			0.11	0.06	0.19	
yes	Rosenberg et al., 2010b	H1	0.17	0.02	0.63	1 / 6
yes	Rapinesi et al., 2015a	H1	0.11	0.02	0.50	1 / 9
yes	Rapinesi et al., 2015b	H1	0.04	0.00	0.40	0 / 12
yes	George et al., 1997	F8	0.14	0.02	0.58	1 / 7
yes	Catafau et al., 2001	F8	0.08	0.01	0.62	0 / 5
yes	Boutros et al., 2002	F8	0.08	0.01	0.41	1 / 12
yes	Yukimasa et al., 2006	F8	0.27	0.13	0.47	7 / 26
yes	Luborzewski et al., 2007	F8	0.29	0.13	0.54	5 / 17
yes			0.20	0.13	0.31	

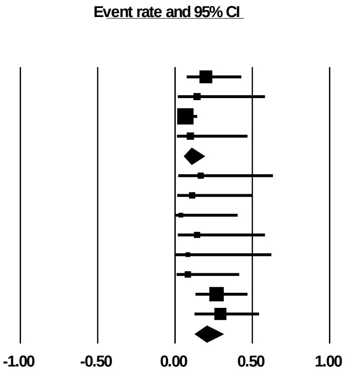

Note. Figures A-B are forest plots of mixed-effects meta-analyses comparing the antidepressant outcomes (remission rates) based on the study designs (RCTs vs. open-label, Figure A) or the therapy type (add-on to antidepressants vs. monotherapy, Figure B) in studies with either coil (F8-coil and H1-coil).

Abbreviations: *CI*, 95% confidence interval; DTMS, deep transcranial magnetic stimulation; F8, figure-of-eight coil (rTMS); H1, H1-coil (DTMS); rTMS, repetitive transcranial magnetic stimulation; Total, sample size per study.

Figure S7. Remission rates: meta-regression analysis

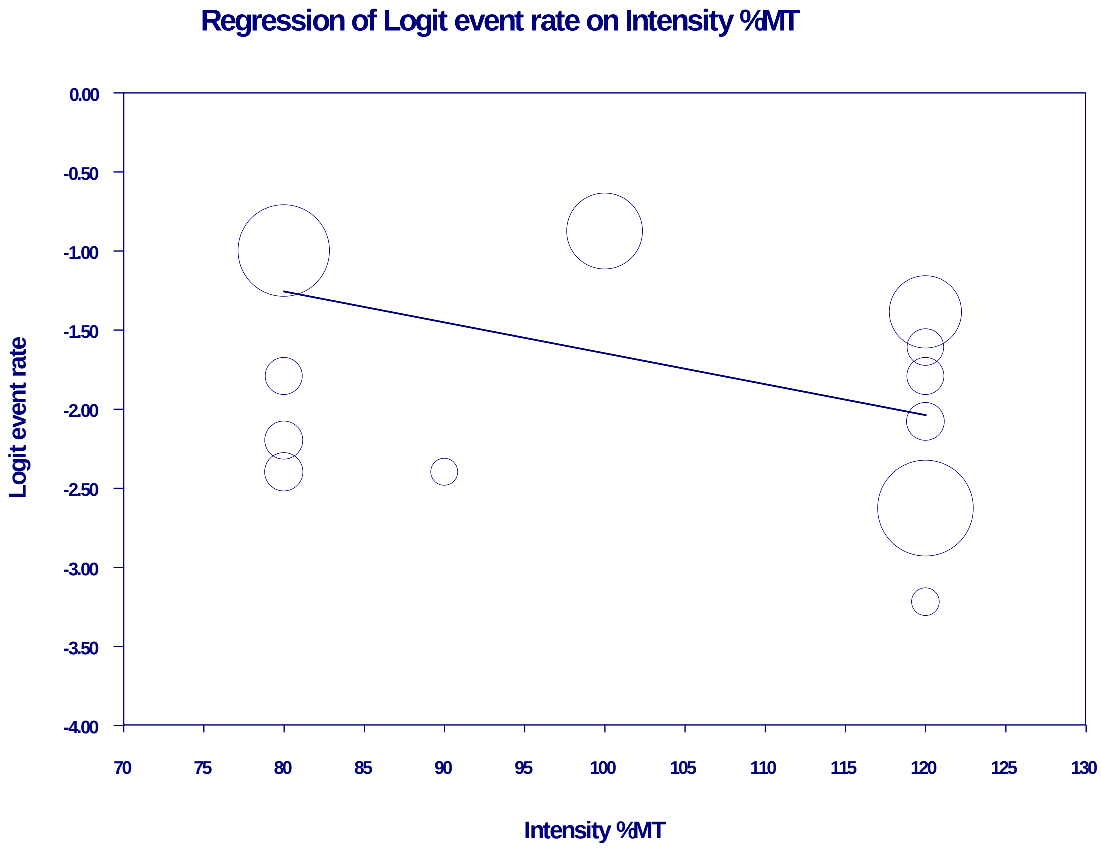

Note. The figure is a scatterplot of random-effects meta-regression. The plot shows the relationships between remission rates expressed as weighted effect size in each study (logit event rate depicted as circles- the larger the circle, the higher the study weight) on the Y-axis and predictor on the X-axis (stimulation intensity, %MT, per study) using data from studies with either coil (F8-coil and H1-coil).

Abbreviations: DTMS, deep transcranial magnetic stimulation; F8, figure-of-eight coil (rTMS); H1, H1-coil (DTMS); %MT, percent of the resting motor threshold; rTMS, repetitive transcranial magnetic stimulation.

Figure S8. Remission rates: publication bias analysis

Classic fail-safe N

Z-value for observed studies	-7.96174
P-value for observed studies	0.00000
Alpha	0.05000
Tails	2.00000
Z for alpha	1.95996
Number of observed studies	12.00000
Number of missing studies that would bring p-value to > alpha	187.00000

Orwin's fail-safe N

Event rate in observed studies	0.15313
Criterion for a 'trivial' event rate	0.10000
Mean event rate in missing studies	0.05000
Number missing studies needed to bring event rate under 0.1	8.00000

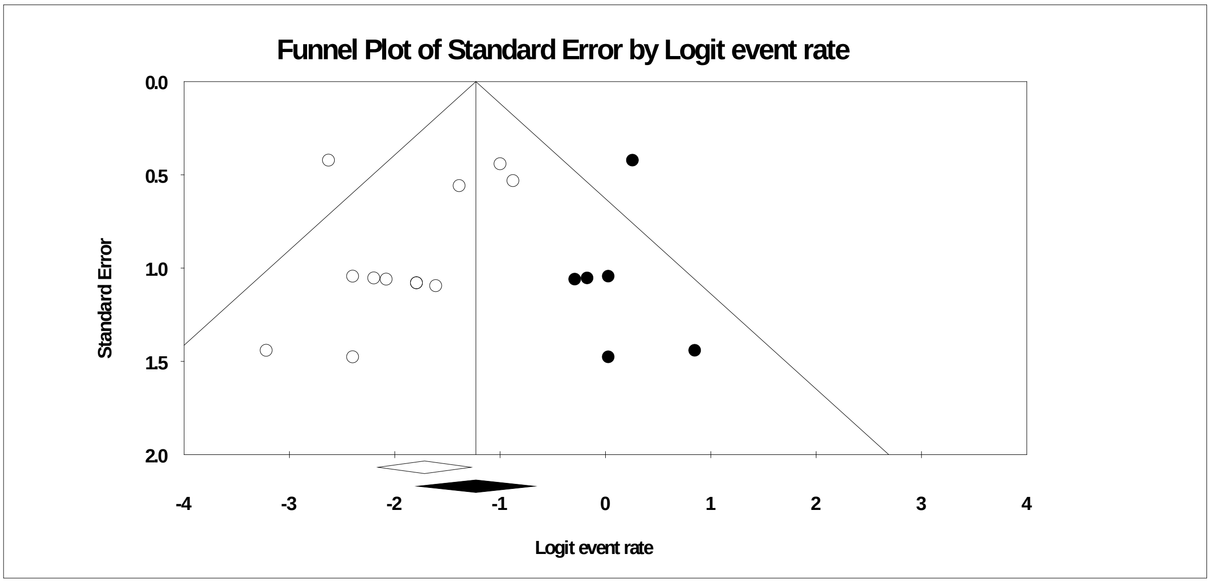

Note. The funnel plot shows a distribution of all effect sizes (the unfilled circles) around the pooled mean weighted effect of all studies (the unfilled diamond). Six studies theoretically missing from the analysis (the filled circles) are required to make the plot symmetric. The interpretation of the analysis including the missing studies (the filled diamond) is the same as without these studies (the unfilled diamond). If the missing studies indicate that publication bias occurred, such bias has no effect on the interpretation of the results of this analysis.

Figure S9. Depression severity: subgroup analysis (H1-coil vs. F8-coil)

A. Open-label studies

Group by Outcome	Study name	Outcome	Hedges's g	Lower limit	Upper limit	p-Value	Total
F8	Catafau et al., 2001	F8	0.31	-0.42	1.03	0.407	5
F8	Bajbouj et al., 2005	F8	0.95	0.53	1.38	0.000	30
F8	Yukimasa et al., 2006	F8	0.78	0.35	1.20	0.000	26
F8	Luborzewski et al., 2007	F8	0.59	0.10	1.09	0.019	17
F8			0.74	0.49	0.98	0.000	
H1	Levkovitz et al., 2009	H1	1.65	0.98	2.31	0.000	20
H1	Rosenberg et al., 2010a	H1	1.48	0.47	2.49	0.004	7
H1	Rosenberg et al., 2010b	H1	1.94	0.65	3.23	0.003	6
H1	Isserles et al., 2011	H1	2.36	1.52	3.20	0.000	20
H1	Harel et al., 2014	H1	1.61	1.04	2.19	0.000	26
H1	Rapinesi et al., 2015a	H1	2.71	1.33	4.09	0.000	9
H1	Rapinesi et al., 2015b	H1	0.84	0.21	1.46	0.009	12
H1			1.67	1.23	2.11	0.000	

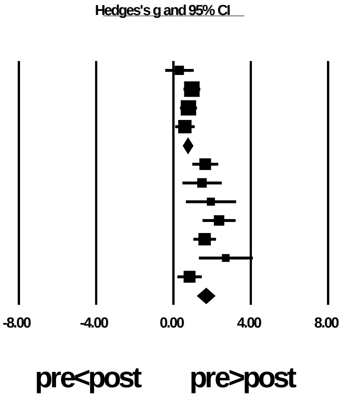

B. Add-on studies

Group by Outcome	Study name	Outcome	Hedges's g	Lower limit	Upper limit	p-Value	Total
F8	George et al., 1997	F8	0.78	0.02	1.54	0.045	7
F8	Catafau et al., 2001	F8	0.31	-0.42	1.03	0.407	5
F8	Garcia-Toro et al., 2001a	F8	1.02	0.45	1.58	0.000	17
F8	Garcia-Toro et al., 2001b	F8	1.28	0.52	2.04	0.001	11
F8	Boutros et al., 2002	F8	0.89	0.26	1.53	0.006	12
F8	Bajbouj et al., 2005	F8	0.95	0.53	1.38	0.000	30
F8	Yukimasa et al., 2006	F8	0.78	0.35	1.20	0.000	26
F8	Luborzewski et al., 2007	F8	0.59	0.10	1.09	0.019	17
F8	Bakim et al., 2012, 80%	F8	1.23	0.51	1.95	0.001	12
F8	Bakim et al., 2012, 110%	F8	1.48	0.66	2.31	0.000	11
F8	Chen et al., 2013	F8	6.40	3.54	9.26	0.000	10
F8			0.96	0.67	1.26	0.000	
H1	Rosenberg et al., 2010b	H1	1.94	0.65	3.23	0.003	6
H1	Isserles et al., 2011	H1	2.36	1.52	3.20	0.000	20
H1	Harel et al., 2014	H1	1.61	1.04	2.19	0.000	26
H1	Rapinesi et al., 2015a	H1	2.71	1.33	4.09	0.000	9
H1	Rapinesi et al., 2015b	H1	0.84	0.21	1.46	0.009	12
H1			1.77	1.11	2.42	0.000	

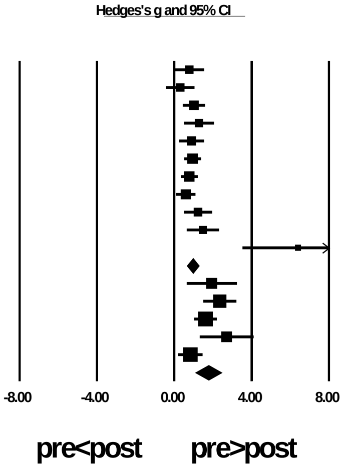

Note. Figures A-B are forest plots of mixed-effects meta-analyses comparing the antidepressant outcomes (depression severity) in studies with F8-coil vs. H1-coil (Figure A: open-label studies; Figure B: studies with patients on concurrent antidepressants).

Abbreviations: *CI*, 95% confidence interval; DTMS, deep transcranial magnetic stimulation; F8, figure-of-eight coil (rTMS); H1, H1-coil (DTMS); Hedges' *g* (effect size), standardised paired difference in means corrected for the sample size; rTMS, repetitive transcranial magnetic stimulation; Total, sample size per study.

Figure S10. Response rates: subgroup analysis (H1-coil vs. F8-coil)

A. Open-label studies

Group by Outcome	Study name	Outcome	Event rate	Lower limit	Upper limit	Total
F8	Catafau et al., 2001	F8	0.40	0.10	0.80	2 / 5
F8	Bajbouj et al., 2005	F8	0.33	0.19	0.52	10 / 30
F8	Yukimasa et al., 2006	F8	0.19	0.08	0.39	5 / 26
F8	Luborzewski et al., 2007	F8	0.35	0.17	0.60	6 / 17
F8			0.30	0.21	0.41	
H1	Levkovitz et al., 2009	H1	0.45	0.25	0.66	9 / 20
H1	Rosenberg et al., 2010a	H1	0.29	0.07	0.67	2 / 7
H1	Rosenberg et al., 2010b	H1	0.50	0.17	0.83	3 / 6
H1	Rapinesi et al., 2015a	H1	0.05	0.00	0.47	0 / 9
H1	Rapinesi et al., 2015b	H1	0.04	0.00	0.40	0 / 12
H1			0.29	0.12	0.53	

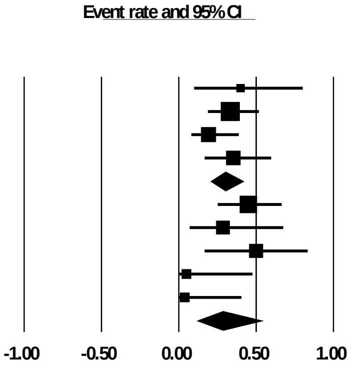

B. Add-on studies

Group by Outcome	Study name	Outcome	Event rate	Lower limit	Upper limit	Total
F8	George et al., 1997	F8	0.14	0.02	0.58	1 / 7
F8	Catafau et al., 2001	F8	0.40	0.10	0.80	2 / 5
F8	Garcia-Toro et al., 2001b	F8	0.36	0.14	0.66	4 / 11
F8	Boutros et al., 2002	F8	0.25	0.08	0.55	3 / 12
F8	Bajbouj et al., 2005	F8	0.33	0.19	0.52	10 / 30
F8	Yukimasa et al., 2006	F8	0.19	0.08	0.39	5 / 26
F8	Luborzewski et al., 2007	F8	0.35	0.17	0.60	6 / 17
F8	Chen et al., 2013	F8	0.70	0.38	0.90	7 / 10
F8			0.33	0.24	0.44	
H1	Rosenberg et al., 2010b	H1	0.50	0.17	0.83	3 / 6
H1	Rapinesi et al., 2015a	H1	0.05	0.00	0.47	0 / 9
H1	Rapinesi et al., 2015b	H1	0.04	0.00	0.40	0 / 12
H1			0.14	0.02	0.62	

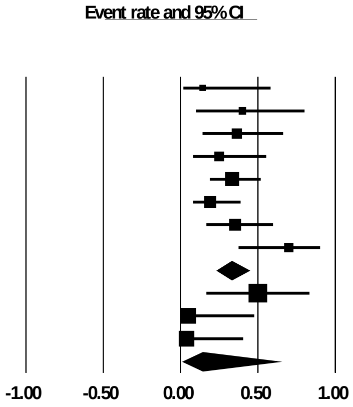

Note. Figures A-B are forest plots of mixed-effects meta-analyses comparing the antidepressant outcomes (response rates) in studies with F8-coil vs. H1-coil (Figure A: open-label studies; Figure B: studies with patients on concurrent antidepressants).

Abbreviations: *CI*, 95% confidence interval; DTMS, deep transcranial magnetic stimulation; F8, figure-of-eight coil (rTMS); H1, H1-coil (DTMS); rTMS, repetitive transcranial magnetic stimulation; Total, sample size per study.

Figure S11. Remission rates: subgroup analysis (H1-coil vs. F8-coil)

A. Open-label studies

Group by Outcome	Study name	Outcome	Event rate	Lower limit	Upper limit	Total
F8	Catafau et al., 2001	F8	0.08	0.01	0.62	0 / 5
F8	Yukimasa et al., 2006	F8	0.27	0.13	0.47	7 / 26
F8	Luborzewski et al., 2007	F8	0.29	0.13	0.54	5 / 17
F8			0.26	0.16	0.41	
H1	Levkovitz et al., 2009	H1	0.20	0.08	0.43	4 / 20
H1	Rosenberg et al., 2010a	H1	0.14	0.02	0.58	1 / 7
H1	Rosenberg et al., 2010b	H1	0.17	0.02	0.63	1 / 6
H1	Rapinesi et al., 2015a	H1	0.11	0.02	0.50	1 / 9
H1	Rapinesi et al., 2015b	H1	0.04	0.00	0.40	0 / 12
H1			0.15	0.08	0.28	

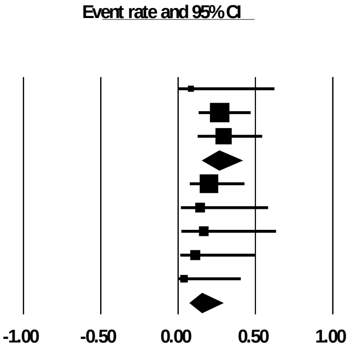

B. Add-on studies

Group by Outcome	Study name	Outcome	Event rate	Lower limit	Upper limit	Total
F8	George et al., 1997	F8	0.14	0.02	0.58	1 / 7
F8	Catafau et al., 2001	F8	0.08	0.01	0.62	0 / 5
F8	Boutros et al., 2002	F8	0.08	0.01	0.41	1 / 12
F8	Yukimasa et al., 2006	F8	0.27	0.13	0.47	7 / 26
F8	Luborzewski et al., 2007	F8	0.29	0.13	0.54	5 / 17
F8			0.23	0.14	0.35	
H1	Rosenberg et al., 2010b	H1	0.17	0.02	0.63	1 / 6
H1	Rapinesi et al., 2015a	H1	0.11	0.02	0.50	1 / 9
H1	Rapinesi et al., 2015b	H1	0.04	0.00	0.40	0 / 12
H1			0.10	0.03	0.30	

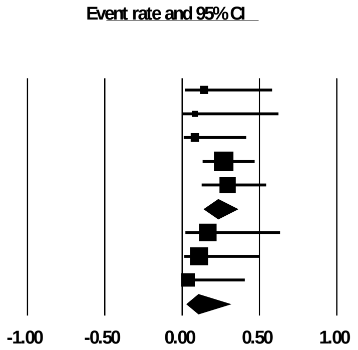

Note. Figures A-B are forest plots of mixed-effects meta-analyses comparing the antidepressant outcomes (remission rates) in studies with F8-coil vs. H1-coil (Figure A: open-label studies; Figure B: studies with patients on concurrent antidepressants).

Abbreviations: *CI*, 95% confidence interval; DTMS, deep transcranial magnetic stimulation; F8, figure-of-eight coil (rTMS); H1, H1-coil (DTMS); rTMS, repetitive transcranial magnetic stimulation; Total, sample size per study.

Table S1. Risk of bias assessment

Study	Random sequence generation (selection bias)	Allocation concealment (selection bias)	Blinding of participants and personnel (performance bias)	Blinding of outcome assessment (detection bias)	Incomplete outcome data (attrition bias)	Selective reporting (reporting bias)	Other bias
**DTMS (H1-coil)**							
Levkovitz et al., 2009 [1]	**H**	**H**	**H**	**H**	L	L	L
Rosenberg et al., 2010a [2]	**H**	**H**	**H**	**H**	L	L	L
Rosenberg et al., 2010b [3]	**H**	**H**	**H**	**H**	L	L	L
Isserles et al., 2011 [4]	**H**	**H**	**H**	**H**	U[2]	L	L
Harel et al., 2014 [5]	**H**	**H**	**H**	**H**	L	L	L
Levkovitz et al., 2015 [6]	L	L	L	L	L	L	L
Rapinesi et al., 2015a [7]	**H**	**H**	**H**	**H**	L	L	L
Rapinesi et al., 2015b [8]	**H**	**H**	**H**	**H**	L	L	L
**rTMS (F8-coil)**							
George et al., 1997 [9]	U[1]	U[1]	L	L	L	L	L
Berman et al., 2000 [10]	U[1]	U[1]	L	L	L	L	L
Catafau et al., 2001 [11]	**H**	**H**	**H**	**H**	L	L	L
Garcia-Toro et al., 2001a [12]	U[1]	U[1]	L	L	U[2]	L	L
Garcia-Toro et al., 2001b [13]	U[1]	U[1]	L	L	U[2]	L	L
Boutros et al., 2002 [14]	L	L	L	L	L	L	L
Bajbouj et al., 2005 [15]	**H**	**H**	**H**	**H**	L	L	L
Yukimasa et al., 2006 [16]	**H**	**H**	**H**	**H**	L	L	L
Luborzewski et al., 2007 [17]	**H**	**H**	**H**	**H**	L	L	L
Bakim et al., 2012a [18]	L	L	L	L	L	L	L
Chen et al., 2013 [19]	U[1]	U[1]	L	L	L	L	L

Note. Coding criteria:

- U[1] indicates that the random sequence generation and/or the allocation concealment were not described in the double-blind randomised controlled-trials with inactive sham groups (both risks are likely to be low)
- U[2] indicates that studies reported outcomes for completers only meaning that the risk of attrition bias is unclear

References

[1] Levkovitz, Y, Harel, EV, Roth, Y, Braw, Y, Most, D, Katz, LN, Sheer, A, Gersner, R, Zangen, A. Deep transcranial magnetic stimulation over the prefrontal cortex: evaluation of antidepressant and cognitive effects in depressive patients. Brain Stimul. 2009;2:188-200.

[2] Rosenberg, O, Shoenfeld, N, Zangen, A, Kotler, M, Dannon, PN. Deep TMS in a resistant major depressive disorder: a brief report. Depress Anxiety. 2010a;27:465-469.

[3] Rosenberg, O, Zangen, A, Stryjer, R, Kotler, M, Dannon, PN. Response to deep TMS in depressive patients with previous electroconvulsive treatment. Brain Stimul. 2010b;3:211-217.

[4] Isserles, M, Rosenberg, O, Dannon, P, Levkovitz, Y, Kotler, M, Deutsch, F, Lerer, B, Zangen, A. Cognitive-emotional reactivation during deep transcranial magnetic stimulation over the prefrontal cortex of depressive patients affects antidepressant outcome. J Affect Disord. 2011;128:235-242.

[5] Harel, EV, Rabany, L, Deutsch, L, Bloch, Y, Zangen, A, Levkovitz, Y. H-coil repetitive transcranial magnetic stimulation for treatment resistant major depressive disorder: an 18-week continuation safety and feasibility study. World J Biol Psychiatry. 2014;15:298-306.

[6] Levkovitz, Y, Isserles, M, Padberg, F, Lisanby, SH, Bystritsky, A, Xia, G, Tendler, A, Daskalakis, ZJ, Winston, JL, Dannon, P, Hafez, HM, Reti, IM, Morales, OG, Schlaepfer, TE, Hollander, E, Berman, JA, Husain, MM, Sofer, U, Stein, A, Adler, S, Deutsch, L, Deutsch, F, Roth, Y, George, MS, Zangen, A. Efficacy and safety of deep transcranial magnetic stimulation for major depression: a prospective multicenter randomized controlled trial. World Psychiatry. 2015;14:64-73.

[7] Rapinesi, C, Bersani, F, Kotzalidis, G, Imperatori, C, Del Casale, A, Di Pietro, S, Ferri, V, Serata, D, Raccah, R, Zangen, A, Angeletti, G, Girardi, P. Maintenance deep transcranial magnetic stimulation sessions is associated with reduced depressive relapses in patients with unipolar or bipolar depression. Front Neurol. 2015a;6:16.

[8] Rapinesi, C, Curto, M, Kotzalidis, GD, Del Casale, A, Serata, D, Ferri, VR, Di Pietro, S, Scatena, P, Bersani, FS, Raccah, RN, Digiacomantonio, V, Ferracuti, S, Bersani, G, Zangen, A, Angeletti, G, Girardi, P. Antidepressant effectiveness of deep transcranial magnetic stimulation (dTMS) in patients with major depressive disorder (MDD) with or without alcohol use disorders (AUDs): a 6-month, open label, follow-up study. J Affect Disord. 2015b;174:57-63.

[9] George, M, Wassermann, EM, Williams, WE, Kimbrell, TA, Little, JT, Hallett, M, Post, RM. Mood improvement following daily left prefrontal repetitive transcranial magnetic stimulation in patients with depression: A placebo-controlled crossover trial. Am J Psychiatry. 1997;154:1752-1756.

[10] Berman, RM, Narasimhan, M, Sanacora, G, Miano, AP, Hoffman, RE, Hu, XS, Charney, DS, Boutros, NN. A randomized clinical trial of repetitive transcranial magnetic stimulation in the treatment of major depression. Biol Psychiatry. 2000;47:332-337.

[11] Catafau, AM, Perez, V, Gironell, A, Martin, JC, Kulisevsky, J, Estorch, M, Carrió, I, Alvarez, E. SPECT mapping of cerebral activity changes induced by repetitive transcranial magnetic stimulation in depressed patients: A pilot study. Psychiatry Res Neuroimaging. 2001;106:151-160.

[12] Garcia-Toro, M, Mayol, A, Arnillas, H, Capllonch, I, Ibarra, O, Crespí, M, Micó, J, Lafau, O, Lafuente, L. Modest adjunctive benefit with transcranial magnetic stimulation in medication-resistant depression. J Affect Disord. 2001a;64:271-275.

[13] García-Toro, M, Pascual-Leone, A, Romera, M, González, A, Micó, J, Ibarra, O, Arnillas, H, Capllonch, I, Mayol, A, Tormos, JM. Prefrontal repetitive transcranial magnetic stimulation as add on treatment for depression. J Neurol Neurosurg Psychiatry. 2001b;71:546-548.

[14] Boutros, NN, Gueorguieva, R, Hoffman, RE, Oren, DA, Feingold, A, Berman, RM. Lack of a therapeutic effect of a 2-week sub-threshold transcranial magnetic stimulation course for treatment-resistant depression. Psychiatry Res. 2002;113:245-254.

[15] Bajbouj, M, Brakemeier, E-L, Schubert, F, Lang, UE, Neu, P, Schindowski, C, Danker-Hopfe, H. Repetitive transcranial magnetic stimulation of the dorsolateral prefrontal cortex and cortical excitability in patients with major depressive disorder. Exp Neurol. 2005;196:332-338.

[16] Yukimasa, T, Yoshimura, R, Tamagawa, A, Uozumi, T, Shinkai, K, Ueda, N, Tsuji, S, Nakamura, J. High-frequency repetitive transcranial magnetic stimulation improves refractory depression by influencing catecholamine and brain-derived neurotrophic factors. Pharmacopsychiatry. 2006;39:52-59.

[17] Luborzewski, A, Schubert, F, Seifert, F, Danker-Hopfe, H, Brakemeier, E-L, Schlattmann, P, Anghelescu, I, Colla, M, Bajbouj, M. Metabolic alterations in the dorsolateral prefrontal cortex after treatment with high-frequency repetitive transcranial magnetic stimulation in patients with unipolar major depression. J Psychiatr Res. 2007;41:606-615.

[18] Bakim, B, Uzun, UE, Karamustafalioglu, O, Ozcelik, B, Alpak, G, Tankaya, O, Cengiz, Y, Yavuz, BG. The combination of antidepressant drug therapy and high-frequency repetitive transcranial magnetic stimulation in medication-resistant depression. Klin Psikofarmakol B. 2012;22:244-253.

[19] Chen, S-J, Chang, C-H, Tsai, H-C, Chen, S-T, Lin, CC. Superior antidepressant effect occurring 1 month after rTMS: add-on rTMS for subjects with medication-resistant depression. Neuropsychiatr Dis Treat. 2013;9:397-401.
